# Practice-Level Spending Variation for Radiation Treatment Episodes Among Older Adults With Cancer

**DOI:** 10.1001/jamahealthforum.2025.1952

**Published:** 2025-07-18

**Authors:** Miranda B. Lam, Mary Beth Landrum, J. Michael McWilliams, Benjamin Buzzee, Alexi A. Wright, Nancy L. Keating, Bruce E. Landon

**Affiliations:** 1 Department of Radiation Oncology, Brigham and Women’s Hospital, Dana-Farber Cancer Institute, Boston, Massachusetts; 2 Department of Health Care Policy, Harvard Medical School, Boston, Massachusetts; 3 Division of General Internal Medicine and Primary Care, Department of Medicine, Brigham and Women’s Hospital, Boston, Massachusetts; 4 Department of Medical Oncology, Dana-Farber Cancer Institute, Boston, Massachusetts; 5 Center for Psycho-Oncology and Palliative Care Research, Dana-Farber Cancer Institute, Boston, Massachusetts; 6 Division of General Medicine, Beth Israel Deaconess Medical Center, Boston, Massachusetts

## Abstract

**Importance:**

Radiation treatments are an essential but expensive component of cancer care.

**Objective:**

To elucidate trends in radiation spending and identify factors associated with practice-level variations across the US health care system to inform alternative payment model design.

**Design, Setting, and Participants:**

This population-based cross-sectional study analyzed fee-for-service Medicare beneficiaries from 2009 to 2020. Patients were continuously enrolled in fee-for-service Medicare Parts A and B during the 1 year prior through 28 days after a radiation treatment episode. Data were analyzed from January 2023 to September 2024.

**Exposures:**

Medicare beneficiaries with cancer who received radiation therapy.

**Main Outcomes and Measures:**

Radiation treatment–specific standardized spending and utilization during 90-day treatment episodes were examined and characterized by radiation type (conformal, intensity modulated, stereotactic, proton, or brachytherapy) and number of fractions. Linear regression models with practice random effects to understand practice-level variation in standardized radiation spending were estimated. Variables were added to adjust for year, patient demographics, cancer type, geography, radiation technology, and number of fractions per episode.

**Results:**

From 2009 to 2020, 1 898 864 beneficiaries with cancer (mean [SD] age, 74 [8.4] years; 48.5% female) initiated 2 149 385 radiation treatment episodes at 2150 practices. Mean (SD) 90-day standardized radiation treatment–specific spending was $13 683 ($8628). Practice-level per-episode radiation-specific spending variation was high (SD after adjusting for year, $4121). It remained high even after adjusting for patient demographic characteristics, cancer type, geography, radiation technology, and number of fractions (SD, $1487). From 2009 to 2020, unadjusted per-episode standardized radiation-specific spending increased slightly from $12 978 to $13 689 (*P* = .04). During this time, the median (IQR) number of fractions per episode decreased from 25 (10-33) to 16 (5-29) (*P* < .001), while the proportion of radiation episodes using intensity-modulated or proton radiation treatment increased (from 5% to 18% and 0.4% to 2%, respectively [both *P* < .001]) and use of conformal radiation treatment decreased from 61% to 38% (*P* < .001).

**Conclusions and Relevance:**

In this cross-sectional study, there was substantial variation in practice-level radiation spending and number of fractions for older patients with cancer undergoing radiation treatment both within and across health care markets. This practice-level variation suggests that there may be opportunities for savings under population-based payment models.

## Key Points

**Question:**

What were the trends and practice-level variations in radiation treatment spending among Medicare beneficiaries with cancer from 2009 to 2020?

**Findings:**

In this cross-sectional study of 1.9 million fee-for-service Medicare beneficiaries across the US receiving radiation therapy, considerable variation in standardized radiation spending was identified at the practice level. This variation persisted after adjusting for factors such as year, patient demographics, cancer type, geography, radiation technology, and number of fractions.

**Meaning:**

The practice-level variation in standardized radiation-specific spending that persisted in local health care markets suggests that there may be opportunities for savings under population-based payment models.

## Teaser

This cross-sectional study aims to elucidate trends in radiation spending and identify factors associated with practice-level variations across the US health care system to inform alternative payment model design.

## Introduction

Alternative payment models (APMs), which link quality and spending targets to reimbursement, have become a cornerstone of US efforts to improve quality while controlling health care spending, including in oncology. Launched in 2016, the Centers for Medicare & Medicaid Services (CMS) Oncology Care Model (OCM) was a voluntary, episode-based payment model for patients with cancer who were undergoing chemotherapy. OCM was associated with lower episode payments, but the savings did not cover the costs of CMS payments to the participating practices.^1,2^ CMS launched the follow-up Enhancing Oncology Model in July 2023.^3^ CMS also previously proposed a mandatory, randomized, prospective radiation oncology APM (RO APM), a 90-day episode-based payment model for patients undergoing radiation treatment. Although the RO APM in its current form will not move forward,^4^ future implementation of APMs in radiation oncology are expected. With more than half of patients with cancer receiving radiation treatment as part of their cancer care, APMs for radiation oncology have the potential to meaningfully affect cancer care delivery.

Prior literature, including in oncology, has demonstrated substantial differences in health care spending across and within geographic regions, and these spending differences are not associated with consistently better quality, access to care, or improved outcomes or survival.^5,6,7,8,9,10^ However, little is known about variation in radiation oncology spending across US practices. Furthermore, although radiation technology has improved, allowing for more precise tumor targeting and hypofractionation (fewer fractions [ie, individual radiation treatments] resulting in shorter radiation courses), differences in the uptake of new radiation technologies, and changes in fractionation across practices may impact spending variation. Prior to the initiation of a new payment or delivery model for radiation oncology, it is important to understand current spending patterns for radiation treatment services and how they have changed over time.

In this study, we used comprehensive data from the Medicare program to examine radiation treatment delivery for Medicare beneficiaries. Specifically, we investigated how radiation spending and delivery has evolved over time. Additionally, we assessed the variation in radiation spending across radiation oncology practices in the US and assessed whether variation is explained by patient, practice, or geographic differences or by differences in the type or number of fractions delivered.

## Methods

### Data Source and Patient Population

We used 100% Medicare data for fee-for-service Medicare beneficiaries with cancer from 2009 to 2020 to examine spending and care for 90-day radiation treatment episodes. Episodes were initiated from January 1, 2009, through October 1, 2020, and ended by December 31, 2020. We identified beneficiaries who had at least 1 inpatient stay or 2 outpatients visits (≥30 days apart) with a primary *International Classification of Diseases, Ninth Revision*/*International Statistical Classification of Diseases and Related Health Problems, Tenth Revision* diagnosis code for cancer. Cancer type was determined by the primary diagnosis code associated with the radiation simulation, categorized into 15 cancer types based on the proposed RO APM (eTable 1 in Supplement 1)^4^ and other cancer types. Patients were continuously enrolled in fee-for-service Medicare Parts A and B during the 1 year prior through 28 days after the radiation treatment episode. Radiation treatment episodes required the presence of a radiation oncology simulation code (episode initiation date), followed by a treatment delivery code within 28 days (eTable 2 in Supplement 1). Patients could initiate additional radiation therapy episodes if they had a new simulation and radiation therapy that occurred at least 28 days after completion of the initial 90-day radiation episode.^4^ Radiation episodes were attributed to a practice (defined based on tax identification number) based on the plurality of radiation treatment delivery codes during the 90-day episode period. We restricted this analyses to the 99.5% of patients assigned to practices with at least 20 assigned radiation episodes in a year.

This study was approved by the institutional review board at Harvard Medical School, which also waived the requirement for obtaining informed consent because the study was deemed minimal risk and used existing available data not collected for this study. We followed the Strengthening the Reporting of Observational Studies in Epidemiology (STROBE) reporting guidelines.

### Radiation Treatment Spending

The primary outcome was radiation treatment–specific spending during the 90-day radiation episodes, consistent with the proposed RO APM, which summed spending from the radiation treatment delivery codes (ie, fractions) proposed for the RO APM.^4^ To address geographic variation in prices, we calculated standardized payments by determining the median total payment amount for each procedure code across all years (eMethods in Supplement 1). We additionally described spending by radiation technology, including 2- or 3-dimensional (2D/3D) conformal therapy, intensity modulated radiation therapy (IMRT), stereotactic, proton, and brachytherapy.

### Variables

Control variables included cancer type, patient sociodemographic characteristics (age, sex, and race and ethnicity based on Research Triangle Institute race codes [Hispanic, Black, White, and other]), year of episode initiation, and any dual eligibility for Medicare and Medicaid in that year. We characterized treatment location as hospital based or at a freestanding radiation facility based on which Medicare file (outpatient vs carrier) included most of a patient’s radiation delivery codes (inpatient radiation was excluded, similar to the RO APM). We characterized the type of radiation technology for each episode based on the predominant technology (eTable 3 in Supplement 1) and summed the number of radiation delivery fractions in each 90-day episode. We documented patients’ hospital referral regions (HRRs) based on their zip code of residence,^11^ approximating the tertiary referral patterns that are common in radiation oncology. We characterized patients’ hierarchical condition category (HCC) scores using publicly available software (version 22 [CMS]) based on diagnosis codes from the year before the radiation episode. There were no missing data other than less than 1% of patients with missing race and ethnicity, who were included in the other race and ethnicity category.

### Statistical Analysis

The unit of analysis was the radiation treatment episode. We calculated unadjusted radiation treatment-specific episode spending by cancer type and over time. To analyze changes over time, we performed linear regressions examining the median number of fractions, technology used, freestanding vs hospital location, and radiation spending. We then estimated a series of linear regression models for radiation spending that included random effects for each practice to understand practice-level variation, sequentially adding controls for year (model 1), sociodemographic characteristics and cancer type (model 2), geographic region (HRR; model 3), type of radiation technology (model 4), number of fractions (model 5), and freestanding vs hospital-based radiation facility (model 6). We repeated these analyses for the 6 most common cancer types for which patients undergo RO treatment (prostate, breast, lung, colorectal, brain metastases, and bone metastases).

A 2-sided *P* < .05 was considered statistically significant. Analyses were conducted using SAS, version 9.4 (SAS Institute). Data were analyzed from January 2023 to September 2024.

## Results

### Patient Characteristics

The study population included 1 898 864 beneficiaries with cancer who underwent 2 149 385 radiation treatment episodes at 2150 practices (eFigure 1 in Supplement 1). The mean (SD) age of the patients with radiation episodes was 74 (8.4) years, 48.5% were female, and 3.9% were Hispanic, 8.6% were Black, 84.0% were White, and 3.5% were another race or ethnicity. The most frequent cancers were breast (17.3%), prostate (16.9%), and lung (16.2%). Most radiation treatment episodes (64.2%) were delivered at a hospital-based practice. Additional episode-level characteristics are summarized in Table 1 and eTable 4 in Supplement 1.

**Table 1.** Baseline Characteristics for Medicare Beneficiaries With Cancer Receiving Radiation Treatment Episodes, 2009-2020

Characteristic	Beneficiaries, %^a^		
	2009-2020	2009	2020^b^
Total No. of episodes	2 149 385	189 114	117 133
Age, mean (SD), y	73.8 (8.4)	73.9 (8.4)	74.0 (7.9)
Race and ethnicity^c^			
Black	8.6	8.6	7.4
Hispanic	3.9	3.7	3.7
White	84.0	85.3	83.9
Other	3.5	2.4	5.0
Documented sex			
Female	48.5	46.2	48.9
Male	51.5	53.8	51.1
Died during 90-d episode	13.8	15.9	11.1
Dual eligibility	10.8	11.3	8.7
Hospital-based radiation	64.2	60.7	68.9
Cancer type			
Anal	0.8	0.6	0.9
Bladder	1.4	1.4	1.3
Bone metastases	10.4	9.6	11.3
Brain metastases	6.8	6.4	6.7
Breast	17.3	16.3	17.4
Cervical	0.5	0.6	0.4
Central nervous system	1.9	2.0	1.6
Colorectal	2.6	3.1	2.0
Head and neck	5.2	5.3	4.8
Lung	16.2	16.3	14.8
Lymphoma	3.0	3.2	2.9
Pancreatic	1.3	1.3	1.4
Prostate	16.9	19.6	17.2
Upper gastrointestinal	2.5	2.5	2.3
Uterine	1.9	1.5	2.8
All others	11.3	10.4	12.2

^a^ All percentages are calculated at the episode level.

^b^ A total of 90 days of data collection was required after the start of a radiation therapy episode; therefore, the final year of analysis (2020) only included three-quarters of a year.

^c^ Based on Research Triangle Institute race codes. Patients with missing race and ethnicity data were included in the other race and ethnicity category.

### Radiation Treatment–Specific Episode Spending

**Figure 1.**
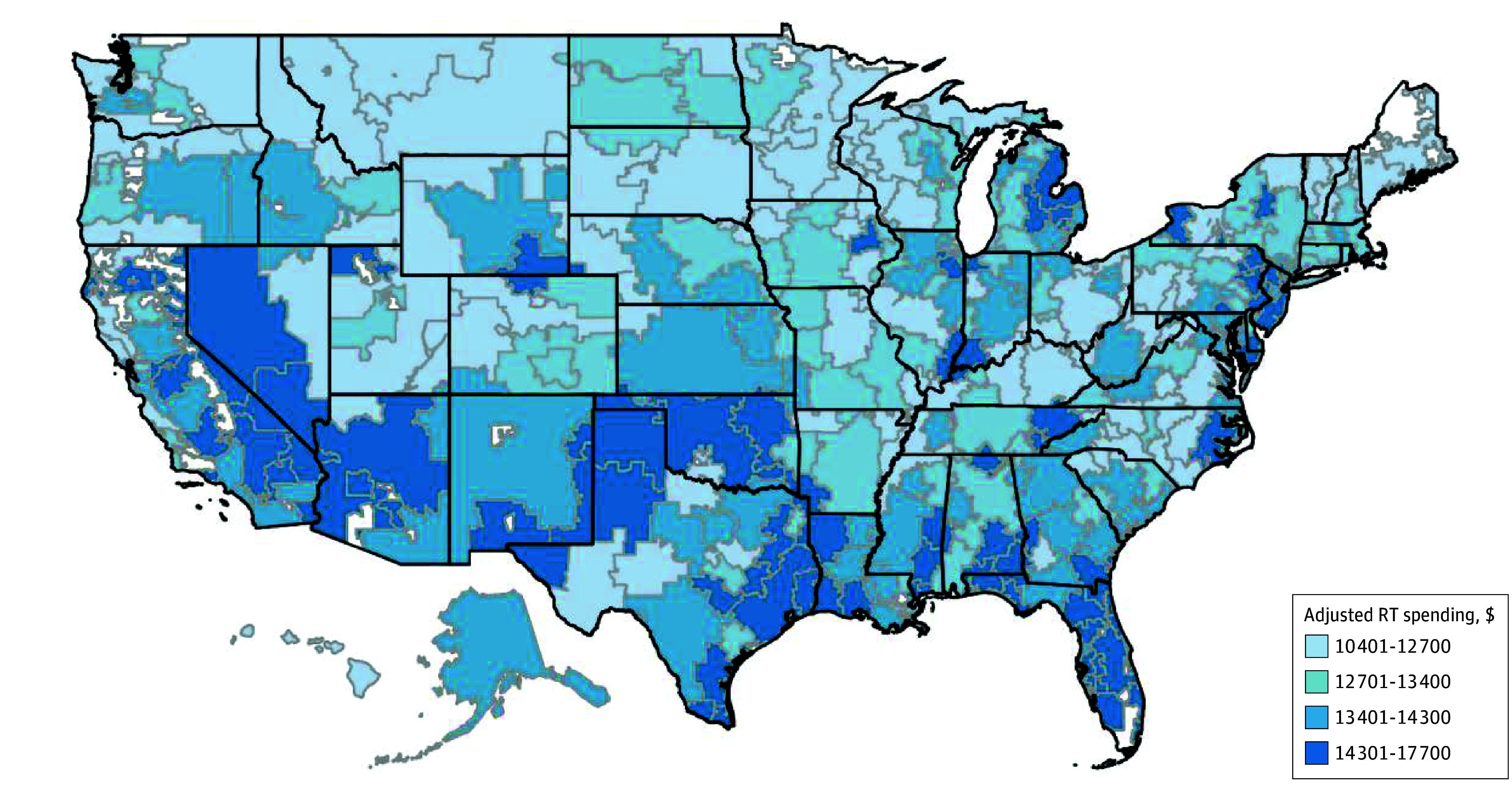
Adjusted Radiation Therapy (RT)–Specific Spending by Hospital Referral Region RT-specific spending is based on standardized payments across all years, adjusted for age, sex, race and ethnicity, Medicaid eligibility, cancer type, and hierarchical condition category.

Mean (SD) unadjusted 90-day standardized radiation treatment–specific spending was $13 683 ($8628), which increased slightly over time, from $12 978 in 2009 to $13 683 in 2020 (*P* = .04; eFigure 2 in Supplement 1). Radiation treatment episode spending was highest for patients with prostate cancer ($24 096), cervical cancer ($18 401), and head and neck cancer ($18 776) (Table 2) and lowest for patients with bone metastases ($6184), brain metastases ($8189), and lymphoma ($8358). Radiation treatment–specific spending by HRR, adjusted for patient age, sex, race and ethnicity, Medicaid eligibility, cancer type, and HCC, demonstrated marked variation across the country (Figure 1).

**Table 2.** Unadjusted Radiation Therapy Spending per Episode by Cancer Type, 2009-2020

Cancer type	Spending, mean (SD), $^a^		
	2009-2020	2009	2020^b^
All cancer types	13 682 (8628)	13 043 (8612)	13 833 (8203)
Anal	17 564 (6032)	14 794 (6176)	18 595 (6055)
Bladder	14 669 (7546)	12 337 (6779)	16 524 (7437)
Bone metastases	6184 (4086)	5686 (3820)	6801 (4412)
Brain metastases	8189 (5267)	6766 (4662)	9791 (5162)
Breast	11 427 (4656)	11 935 (4925)	10 724 (4384)
Cervical	18 401 (9132)	16 206 (8426)	20 843 (9195)
Central nervous system	15 132 (6992)	13 272 (6707)	15 772 (6483)
Colorectal	12 945 (5995)	11 335 (5476)	14 195 (6349)
Head and neck	18 776 (7227)	16 796 (7156)	20 213 (7652)
Lung	12 823 (6781)	11 113 (6340)	13 913 (6534)
Lymphoma	8358 (4743)	7982 (4347)	9096 (5198)
Pancreatic	14 409 (6241)	12 399 (6091)	14 870 (5789)
Prostate	24 096 (9050)	23 006 (9282)	22 432 (8616)
Upper gastrointestinal	14 140 (6662)	11 473 (5720)	15 941 (6919)
Uterine	14 177 (7667)	14 231 (6851)	12 906 (7681)
All others	11 091 (7201)	9884 (6764)	11 899 (7208)

^a^ Spending was calculated using standardized payments across all years.

^b^ A total of 90 days of data collection was required after the start of a radiation therapy episode; therefore, the final year of analysis (2020) only included three-quarters of a year.

### Type of Radiation Treatment Technology, Number of Fractions, and Location of Radiation Treatment

**Figure 2.**
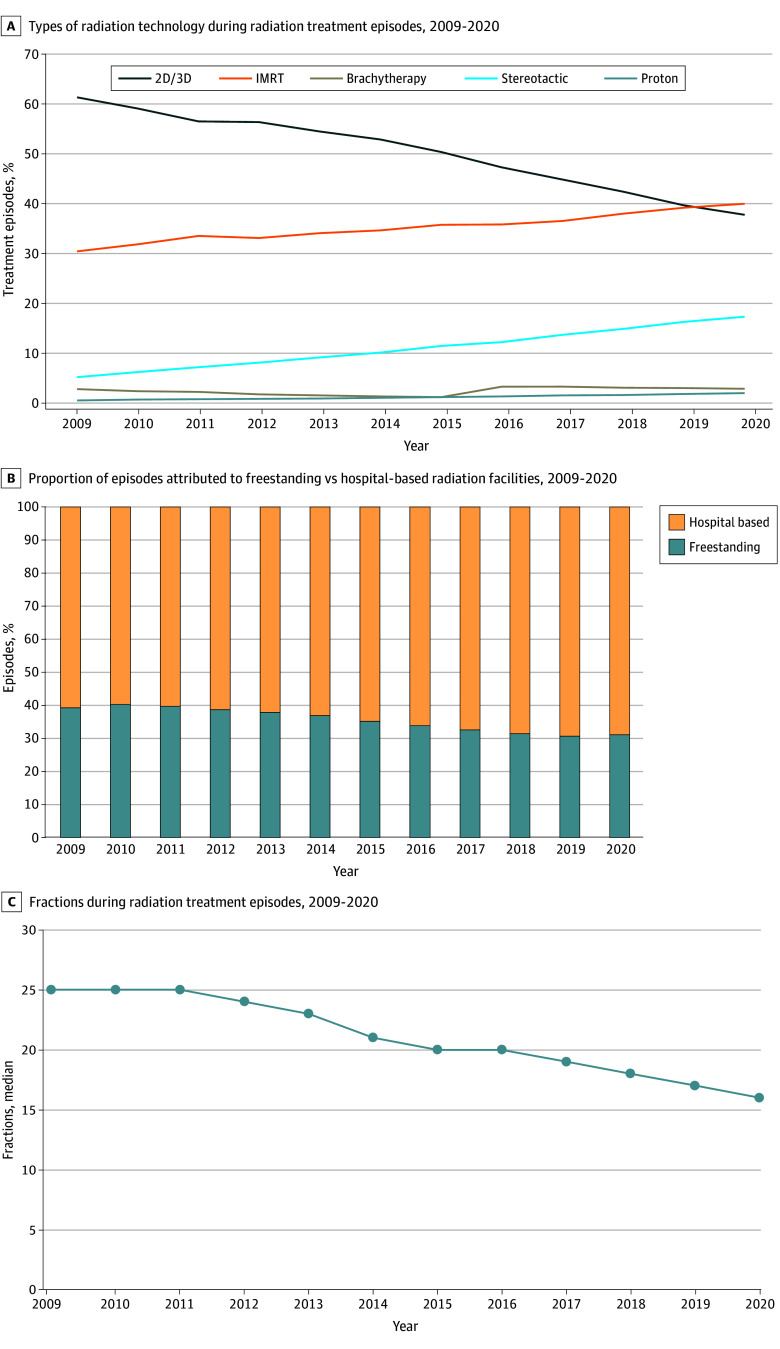
Trends Over Time in Spending per Radiation Therapy Episode by Radiation Type of Technology, Proportion of Episodes Delivered at Freestanding vs Hospital-Based Facilities, and Median Number of Fractions per Episode 2D/3D Indicates 2- or 3-dimensional conformal therapy; IMRT, intensity modulated radiation therapy.

The use of different radiation treatment technologies has changed over time (Figure 2A and eFigure 7 in Supplement 1). From 2009 to 2020, the use of 2D/3D conformal radiation fell from 61% to 38% (*P* < .001), while IMRT increased from 5% to 18% (*P* < .001) and proton radiation increased from 0.4% to 2% (*P* < .001). Brachytherapy use remained at 3% (*P* = .20). The number of practices using only IMRT and only stereotactic RT was small and decreased over time (eTables 6 and 7 in Supplement 1). Treatment at hospital-based facilities increased from 61% of episodes in 2009 to 69% in 2020 (Figure 2B). The median (IQR) number of fractions per episode decreased over time, from 25 (10-33) fractions in 2009 to 16 (5-29) fractions in 2020 (*P* < .001; Figure 2C). There were similar decreases in the median number of fractions for most cancer types between 2009 and 2020, except for anal, colorectal, and upper GI cancers, which remained stable (28, 27, and 25 respectively), and cervical cancer, which increased (25 to 29; eFigure 3 in Supplement 1).

### Practice-Level Variation in Episode Spending and Number of Fractions

**Figure 3.**
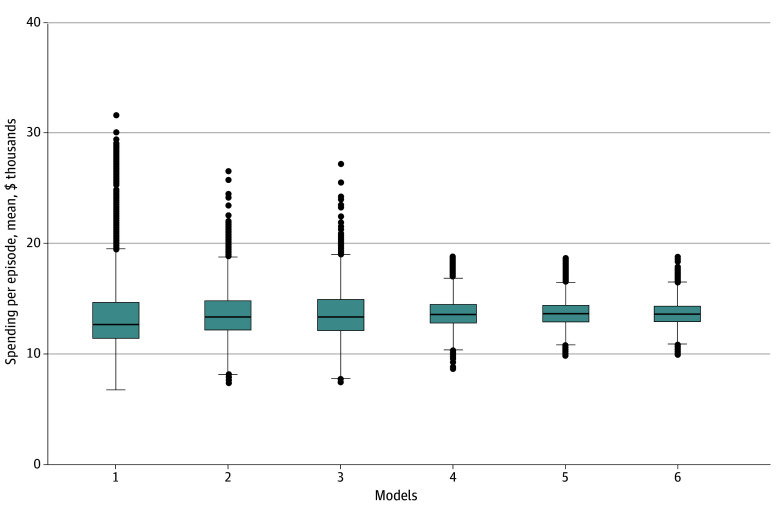
Adjusted Practice-Level Radiation Therapy–Specific Spending per Episode for All Patients With Cancer Receiving Radiation Therapy, 2009-2020 Model 1 was unadjusted (only adjusted for year); model 2 was adjusted for year, age, sex, dual eligibility, hierarchical chronic conditions, and cancer type; model 3 was adjusted for year, age, sex, dual eligibility, hierarchical chronic conditions, cancer type, and hospital referral region; model 4 was adjusted for year, age, sex, dual eligibility, hierarchical chronic conditions, cancer type, hospital referral region, and radiation technology type; model 5 was adjusted for year, age, sex, dual eligibility, hierarchical chronic conditions, cancer type, hospital referral region, radiation technology type, and number of fractions; and model 6 was adjusted for year, age, sex, dual eligibility, hierarchical chronic conditions, cancer type, hospital referral region, radiation technology type, number of fractions, and freestanding vs hospital-based radiation facility. Outliers (<1%, >99%) were suppressed for the figure. Spending was calculated using standardized payments across all years. The bottom of the box represents the 25th percentile, the middle line in the box is the median, and the top of the box is the 75th percentile. The top whisker extends to the largest value no more than 1.5 times the IQR from the top of the box, and the bottom whisker extends to the lowest value no less than 1.5 times the IQR from the bottom of the box. The outlier dots are any values beyond the whiskers.

In sequential models assessing the primary associations of practice-level variation with standardized radiation-specific episode spending adjusting for year only, variation was high, as demonstrated by large SDs (model 1: SD, $4121; Figure 3). About one-third of the variation observed in model 1 was accounted for by differences in patient sociodemographic factors, cancer type, and geographic region (model 3: SD, $2843). Practice-level variation did not meaningfully change after including geographic region (model 2: SD, $2756). Adjusting for technology type (model 4: SD, $1781) and number of fractions (model 5: SD, $1467) was associated with a further one-third reduction in practice-level variation. Adjusting for hospital vs freestanding radiation facility was not associated with a change in variation (model 6: SD, $1444). Results of sequential models assessing practice-level variation in radiation treatment spending in 2009 and 2020 were similar to the overall results (eFigure 4 in Supplement 1).

When investigating practice-level variation separately for prostate cancer, breast cancer, lung cancer, colorectal cancer, bone metastases, and brain metastases, relatively little variation (0%-5%) was explained by patient sociodemographic factors, cancer type, or region. Technology type and number of fractions was associated with a reduction in practice-level variation by approximately half, indicating substantial practice-level variation in radiation spending after all adjustments (eFigure 5 and eTable 5 in Supplement 1). Finally, variation in practice-level number of fractions was also investigated. There was substantial variation in the number of fractions per episode across practices (mean [SD], 21.0 [5.6] in the unadjusted base model; 21.0 [4.2] after adjustment for patient demographics, cancer type, and geographic area; and 21.0 [4.2] after adjustment for technology type; eFigure 6 in Supplement 1).

## Discussion

In this national population of fee-for-service Medicare beneficiaries with cancer undergoing radiation treatment from 2009 to 2020, we observed substantial variation in practice-level spending that persisted over time. One-third of practice-level variation remained after all adjustments, with observed variation reflecting variation within rather than between markets. Standardized radiation-specific spending increased slightly over time. Over time, the use of new modalities such as IMRT, stereotactic, and proton radiation treatment increased, while the use of 2D/3D conformal radiation treatment decreased. Practice-level variation in radiation treatment spending and in the number of fractions per episode within a market remained high, even after accounting for patient characteristics, cancer type, geography, and technology type. These findings provide a comprehensive analysis of radiation-specific spending and variation across the US. The substantial practice-level variation, which persisted within health care markets, suggests that there may be opportunities for savings for APMs targeting radiation oncology practices. Even outside the context of a focused APM, however, these results suggest that accountable care and other risk-bearing organizations focused on general populations also might achieve savings by identifying and referring patients to practices that optimize the value of radiation delivery. An important component of all APMs, including in radiation oncology, also requires understanding of the quality of care, which was not assessed in this study, to ensure that savings do not come at the expense of quality.

With cancer care costs projected to reach $246 billion by 2030,^12^ oncology-focused APMs have garnered considerable interest from policymakers and payers.^1,2^ Given the lack of savings associated with OCM and high spending on other cancer-directed treatments,^13,14^ CMS and other payers have explored opportunities in radiation oncology. Radiation treatments are traditionally reimbursed per fraction; such volume-based reimbursements provide financial incentives that might unintentionally promote longer treatment courses rather than shorter ones (hypofractionation) that have been shown to be equally efficacious in certain cancer types, which could improve patient convenience and lessen financial strain. APMs that eliminate these incentives for more care thus have potential to improve value. While the CMS RO APM model will not proceed as originally proposed due to concerns raised by various stakeholders,^15,16,17,18,19^ future APMs in radiation oncology are expected. For instance, the American Society for Radiation Oncology recently proposed an APM (Radiation Oncology Case Rate) that builds on the prior CMS model.^20^

The generally stable radiation spending over time appears to reflect 2 offsetting factors: (1) a decrease in the median number of fractions per episode and (2) an increase in the use of advanced technologies for treatment delivery (IMRT, stereotactic, and proton),^21^ which are more expensive and allow for shorter courses of radiation to be delivered more safely. The increase in IMRT—now the standard of care for several cancer types due to its precise targeting—is unsurprising.^22,23^ Stereotactic body radiation therapy (SBRT) allows for high doses of radiation to be given over a shorter course. Recent evidence has suggested that SBRT for patients with oligometastatic disease may improve survival,^24,25^ but further trials are needed to confirm these results. SBRT can also safely re-irradiate previously treated areas in the body. In contrast, although proton radiation treatment has advantages for pediatric or re-irradiation cases, evidence of benefit is mixed for frequently diagnosed cancers in older adults, such as prostate cancer.^26^ Newer technologies are typically more expensive, and episode-based payment models could help limit overuse of radiation technology not proven beneficial. However, in clinical cases for which newer technology has proven benefits, it will be important to ensure that episode-based payments do not incentivize practices to offer fewer effective treatments for patients. This highlights both the challenge and importance of minimizing the potential unintended consequences of APMs for a specialty that uses advanced technology.

The present findings confirm a meaningful decrease in the median number of fractions for patients with cancer undergoing radiation treatment, from 25 in 2009 to 16 in 2020. This was observed across most cancers, including prostate, breast, and lung cancer. Several clinical trials in breast and prostate cancer have shown that for certain patients, hypofractionated radiation treatment has similar outcomes and toxic effects as longer courses of radiation.^27,28,29,30^ For lung cancer, the use of stereotactic therapy is associated with improved tumor targeting, local control, and adverse effects, allowing patients who are medically inoperable or decline surgery for early-stage lung cancer to be safely treated with 15 or fewer fractions of hypofractionated or stereotactic lung radiation therapy for curative treatment.^31,32^ Despite the overall decline in the number of fractions over time, we observed substantial local variation in the number of fractions used, which suggests one potential area of focus for increasing the value of radiation treatment.

We found that variation in radiation treatment spending was associated with several different factors. About one-third of the variation was associated with patient demographics and cancer type, with geography having little impact on the variation. Another one-third of the variation was associated with the type of radiation technology and the number of fractions. The remaining one-third of the practice-level variation was associated with factors that we could adjust for using claims data. These results have several important implications. The variation associated with radiation technology and number of fractions highlights the need for research to empirically test radiation strategies for specific cancers. For instance, ongoing randomized studies evaluating proton radiation among many different cancer types are important and necessary in a field that has shown improved cancer outcomes and toxic effects through innovation.^33^ Additionally, offering fewer fractions may not be better or evidence based for all cancer types; hence, efforts to lower spending on cancer care must not lead to less appropriate or nonguideline-concordant care. Second, the present findings that a third of practice-level variation in radiation spending remains after adjusting for patient characteristics, cancer type, region, radiation technology type, and number of fractions suggest that APMs, which generally function in local areas, may promote more consistent practice patterns and potentially decreased spending,^7^ although additional research is needed to understand the drivers of this variation. A prior study on patient-level variation in spending on radiation therapy found that 43% to 61% of variation was not explained by practice type, geography, or radiation oncologist.^34^ The present study extends these findings by focusing on practice-level spending and considering implications for potential APMs.

### Limitations

This study has several limitations. First, the data lacked information about patients’ performance status, cancer stage, intent of treatment (palliative or curative, aside from those being treated for brain or bone metastases), and recommended treatment, and we were unable to verify the appropriateness of the radiation technology or fractionations used. Second, we could not assess whether lower-spending practices were providing equivalent care to higher-spending practices. However, proposed APMs use similar methodology. Innovative methodologies that incorporate clinical data, including natural language processing or standardization of electronic health record data elements, may provide future opportunities for assessing quality. Third, we assigned each patient to a single radiation modality based on the majority of treatments, yet some patients received a combination of therapies (eg, a patient with cervical cancer may receive 3D or IMRT radiation to the pelvis, followed by brachytherapy). However, we accounted for all radiation spending, irrespective of the technology group assigned. Fourth, Medicare prices were not inflation adjusted; however, prices paid by Medicare are also not inflation adjusted and have not kept up with inflation. Additionally, the standardized spending measure is based on mean total spending across years, addressing any time-related differences in spending. Fifth, since billing data are imperfect, there may be some misclassification of cancer type (eg, bone metastases from lung cancer could potentially be coded as lung cancer instead of bone metastases). Finally, this study focused on fee-for-service Medicare beneficiaries and results may not generalize to younger patients or those enrolled in Medicare Advantage, for which enrollment doubled during the study period.

## Conclusions

In this cross-sectional study examining 90-day radiation treatment over time, we found greater use of high-cost advanced technology (eg, IMRT, stereotactic radiation) offset by a decline in the mean number of fractions per radiation treatment episode. We observed marked area-level variation in radiation treatment episode-based spending and number of fractions per episode across the US, with a third of the variation associated with practice-level factors and not patient demographics, cancer type, geographic location, radiation technology, or number of fractions. This variation across practices suggests that there may be opportunities for savings in a future value-based payment model for radiation oncology.

## Notes

Supplement 1.
eMethods
**eTable 1. **Cancer Diagnoses Codes **eTable 2. **Radiation Treatment HCPCS/CPT Codes to Define a Radiation Episode **eTable 3.** Radiation Treatment HCPCS/CPT Codes by Type of Technology **eTable 4.** Cancer Patient Mortality during 90-Day Radiation Treatment Episodes **eTable 5.** Practice-level Adjusted Radiation Spending, Additional Models **eFigure 1.** Flowchart for Patient Population **eFigure 2.** Unadjusted Standardized Spending for Radiation Therapy-Specific Services for 90-Day Radiation Therapy Episodes Over Time (2009-2020) **eFigure 3.** Median Number of Radiation Fractions Overall and by Cancer Type, 2009 and 2020 **eFigure 4.** Adjusted Variation in Per-Practice Radiation Spending for All Cancer Patients Receiving RT, 2009 and 2020 **eFigure 5.** Adjusted Variation in Per-Practice Radiation Spending by Common Cancer Types **eFigure 6.** Adjusted Variation in Per-Practice Fraction Number for All Cancer Patients Receiving Radiation Treatments **eFigure 7. **Types of Radiation Technology by Common Cancer Types **eTable 6.** Practice-Level Proportion of Only IMRT or Only Stereotactic RT Use Over Time **eTable 7. **Number of Practices Utilizing Only IMRT or Only Stereotactic RT Over Time

Supplement 2.
Data Sharing Statement

## References

[1] Evaluation of the Oncology Care Model: performance periods 1-9. Centers for Medicare & Medicaid Services. June 2023. Accessed July 13, 2023. https://www.cms.gov/priorities/innovation/data-and-reports/2023/ocm-evaluation-pp1-9

[2] Keating NL, Jhatakia S, Brooks GA, ; Oncology Care Model Evaluation Team. Association of participation in the Oncology Care Model with Medicare payments, utilization, care delivery, and quality outcomes. JAMA. 2021;326(18):1829-1839. doi:10.1001/jama.2021.17642 34751709 PMC8579232

[3] Kocher RP, Adashi EY. A new approach to cancer bundled payments in Medicare—the Enhancing Oncology Model. JAMA Health Forum. 2023;4(1):e224904. doi:10.1001/jamahealthforum.2022.4904 36662504

[4] Radiation Oncology Model. Centers for Medicare & Medicaid Services. Accessed May 12, 2023. https://www.cms.gov/priorities/innovation/innovation-models/radiation-oncology-model

[5] Johnson WC, Biniek JF. Sources of geographic variation in health care spending among individuals with employer sponsored insurance. Med Care Res Rev. 2021;78(5):548-560. doi:10.1177/1077558720926095 32633204 PMC8414822

[6] Newhouse JP, Garber AM. Geographic variation in Medicare services. N Engl J Med. 2013;368(16):1465-1468. doi:10.1056/NEJMp1302981 23520983

[7] Clough JD, Patel K, Riley GF, Rajkumar R, Conway PH, Bach PB. Wide variation in payments for Medicare beneficiary oncology services suggests room for practice-level improvement. Health Aff (Millwood). 2015;34(4):601-608. doi:10.1377/hlthaff.2014.0964 25847642

[8] Keating NL, Huskamp HA, Kouri E, . Factors contributing to geographic variation in end-of-life expenditures for cancer patients. Health Aff (Millwood). 2018;37(7):1136-1143. doi:10.1377/hlthaff.2018.0015 29985699 PMC6059805

[9] Keating NL, Landrum MB, Lamont EB, Bozeman SR, McNeil BJ. Area-level variations in cancer care and outcomes. Med Care. 2012;50(5):366-373. doi:10.1097/MLR.0b013e31824d74c0 22437623

[10] McWilliams JM, Dalton JB, Landrum MB, Frakt AB, Pizer SD, Keating NL. Geographic variation in cancer-related imaging: Veterans Affairs health care system versus Medicare. Ann Intern Med. 2014;161(11):794-802. doi:10.7326/M14-0650 25437407 PMC4251705

[11] Research methods. Dartmouth Atlas of Health Care. Accessed November 17, 2024. https://www.dartmouthatlas.org/research-methods/

[12] Mariotto AB, Enewold L, Zhao J, Zeruto CA, Yabroff KR. Medical care costs associated with cancer survivorship in the United States. Cancer Epidemiol Biomarkers Prev. 2020;29(7):1304-1312. doi:10.1158/1055-9965.EPI-19-1534 32522832 PMC9514601

[13] Newcomer LN, Gould B, Page RD, Donelan SA, Perkins M. Changing physician incentives for affordable, quality cancer care: results of an episode payment model. J Oncol Pract. 2014;10(5):322-326. doi:10.1200/JOP.2014.001488 25006221

[14] Bekelman JE, Gupta A, Fishman E, . Association between a national insurer’s pay-for-performance program for oncology and changes in prescribing of evidence-based cancer drugs and spending. J Clin Oncol. 2020;38(34):4055-4063. doi:10.1200/JCO.20.00890 33021865

[15] Meeks SL, Mathews R, Mojica J, Shah AP, Kelly P, Dvorak T. Impact of radiation oncology alternative payment model on community cancer centers. JCO Oncol Pract. 2021;17(12):e1949-e1957. doi:10.1200/OP.21.00298 34460290

[16] Pendyala P, Goglia AG, Young R, Suh JH, Ennis RD. Radiation oncology alternative payment model and large urban academic centers: future implications for patients and providers. JCO Oncol Pract. 2021;17(12):e1968-e1976. doi:10.1200/OP.21.00347 34678044 PMC9810124

[17] Bates JE, Thaker NG, Shah CS, Royce TJ. Geography of the radiation oncology alternative payment model. JCO Oncol Pract. 2021;17(12):770-772. doi:10.1200/OP.21.00304 34705494

[18] Mantz CA, Thaker NG, Pendyala P, . Disproportionate negative impact of the radiation oncology alternative payment model on rural providers: a cost identification analysis of Medicare claims. JCO Oncol Pract. 2021;17(12):e1977-e1983. doi:10.1200/OP.21.00330 34529516

[19] Luh JY, Jones RT, Thaker NG, . An overview of the radiation oncology alternative payment model and impact on practices serving vulnerable populations. J Am Coll Radiol. 2022;19(1 pt A):53-60. doi:10.1016/j.jacr.2021.09.030 34762833

[20] Chollet-Lipscomb C, Luh JY, Mantz C, Milligan M. Radiation Oncology Case Rate Program (ROCR). American Society for Radiation Oncology. July 21, 2023. Accessed April 28, 2024. https://www.astro.org/advocacy/key-issues-8f3e5a3b76643265ee93287d79c4fc40/rocr

[21] Shen X, Showalter TN, Mishra MV, . Radiation oncology services in the modern era: evolving patterns of usage and payments in the office setting for Medicare patients from 2000 to 2010. J Oncol Pract. 2014;10(4):e201-e207. doi:10.1200/JOP.2013.001270 24756145

[22] Hogan JS, Karraker P, Fischer-Valuck BW, . Benchmarking the radiation oncology alternative payment model: changes in Medicare reimbursement for 16 common radiation therapy treatment courses. Pract Radiat Oncol. 2023;13(5):e389-e394. doi:10.1016/j.prro.2023.04.012 37172757

[23] Hogan J, Roy A, Karraker P, . Decreases in radiation oncology Medicare reimbursement over time: analysis by billing code. Int J Radiat Oncol Biol Phys. 2022;114(1):47-56. doi:10.1016/j.ijrobp.2022.05.018 35613687 PMC10077845

[24] Palma DA, Olson R, Harrow S, . Stereotactic ablative radiotherapy for the comprehensive treatment of oligometastatic cancers: long-term results of the SABR-COMET phase II randomized trial. J Clin Oncol. 2020;38(25):2830-2838. doi:10.1200/JCO.20.00818 32484754 PMC7460150

[25] Gillespie EF, Yang JC, Mathis NJ, . Prophylactic radiation therapy versus standard of care for patients with high-risk asymptomatic bone metastases: a multicenter, randomized phase II clinical trial. J Clin Oncol. 2024;42(1):38-46. doi:10.1200/JCO.23.00753 37748124 PMC10730067

[26] Kamran SC, Light JO, Efstathiou JA. Proton versus photon-based radiation therapy for prostate cancer: emerging evidence and considerations in the era of value-based cancer care. Prostate Cancer Prostatic Dis. 2019;22(4):509-521. doi:10.1038/s41391-019-0140-7 30967625

[27] Catton CN, Lukka H, Gu CS, . Randomized trial of a hypofractionated radiation regimen for the treatment of localized prostate cancer. J Clin Oncol. 2017;35(17):1884-1890. doi:10.1200/JCO.2016.71.7397 28296582

[28] Dearnaley D, Syndikus I, Mossop H, ; CHHiP Investigators. Conventional versus hypofractionated high-dose intensity-modulated radiotherapy for prostate cancer: 5-year outcomes of the randomised, non-inferiority, phase 3 CHHiP trial. Lancet Oncol. 2016;17(8):1047-1060. doi:10.1016/S1470-2045(16)30102-4 27339115 PMC4961874

[29] Smith BD, Bellon JR, Blitzblau R, . Radiation therapy for the whole breast: executive summary of an American Society for Radiation Oncology (ASTRO) evidence-based guideline. Pract Radiat Oncol. 2018;8(3):145-152. doi:10.1016/j.prro.2018.01.012 29545124

[30] Gillespie EF, Tringale KR, Bach PB, Bekelman JE. Evaluation of use of shorter radiation regimens for breast and prostate cancer in the US, 2015-2017. JAMA Netw Open. 2020;3(7):e2010519. doi:10.1001/jamanetworkopen.2020.10519 32672827 PMC7366185

[31] Buchberger DS, Videtic GMM. Stereotactic body radiotherapy for the management of early-stage non-small-cell lung cancer: a clinical overview. JCO Oncol Pract. 2023;19(5):239-249. doi:10.1200/OP.22.00475 36800644

[32] Iyengar P, Zhang-Velten E, Court L, . Accelerated hypofractionated image-guided vs conventional radiotherapy for patients with stage II/III non–small cell lung cancer and poor performance status: a randomized clinical trial. JAMA Oncol. 2021;7(10):1497-1505. doi:10.1001/jamaoncol.2021.3186 34383006 PMC8531992

[33] Bekelman JE, Denicoff A, Buchsbaum J. Randomized trials of proton therapy: why they are at risk, proposed solutions, and implications for evaluating advanced technologies to diagnose and treat cancer. J Clin Oncol. 2018;36(24):2461-2464. doi:10.1200/JCO.2018.77.7078 29985746 PMC6366815

[34] Paravati AJ, Boero IJ, Triplett DP, . Variation in the cost of radiation therapy among Medicare patients with cancer. J Oncol Pract. 2015;11(5):403-409. doi:10.1200/JOP.2015.005694 26265172 PMC4575405

