# Supplementary material for: Practice-Level Spending Variation for Radiation Treatment Episodes Among Older Adults With Cancer
Source: JAMA Health Forum. 2025 Jul 18;6(7):e251952. doi: 10.1001/jamahealthforum.2025.1952 (PMC12274980; doi:10.1001/jamahealthforum.2025.1952)
Supplement: Supplement 1. — eMethods eTable 1. Cancer Diagnoses Codes eTable 2. Radiation Treatment HCPCS/CPT Codes to Define a Radiation Episode eTable 3. Radiation Treatment HCPCS/CPT Codes by Type of Technology eTable 4. Cancer Patient Mortality during 90-Day Radiation Treatment Episodes eTable 5. Practice-level Adjusted Radiation Spending, Additional Models eFigure 1. Flowchart for Patient Population eFigure 2. Unadjusted Standardized Spending for Radiation Therapy-Specific Services for 90-Day Radiation Therapy Episodes Over Time (2009-2020) eFigure 3. Median Number of Radiation Fractions Overall and by Cancer Type, 2009 and 2020 eFigure 4. Adjusted Variation in Per-Practice Radiation Spending for All Cancer Patients Receiving RT, 2009 and 2020 eFigure 5. Adjusted Variation in Per-Practice Radiation Spending by Common Cancer Types eFigure 6. Adjusted Variation in Per-Practice Fraction Number for All Cancer Patients Receiving Radiation Treatments eFigure 7. Types of Radiation Technology by Common Cancer Types eTable 6. Practice-Level Proportion of Only IMRT or Only Stereotactic RT Use Over Time eTable 7. Number of Practices Utilizing Only IMRT or Only Stereotactic RT Over Time [file jamahealthforum-e251952-s001.pdf]

Supplemental Online Content

Lam MB, Landrum MB, McWilliams JM, et al. Practice-level spending variation for radiation treatment episodes among older adults with cancer. *JAMA Health Forum*. 2025;6(7):e251952. doi:10.1001/jamahealthforum.2025.1952

eMethods

eTable 1. Cancer Diagnoses Codes

eTable 2. Radiation Treatment HCPCS/CPT Codes to Define a Radiation Episode

eTable 3. Radiation Treatment HCPCS/CPT Codes by Type of Technology

eTable 4. Cancer Patient Mortality during 90-Day Radiation Treatment Episodes

eTable 5. Practice-level Adjusted Radiation Spending, Additional Models

eFigure 1. Flowchart for Patient Population

eFigure 2. Unadjusted Standardized Spending for Radiation Therapy-Specific Services for 90-Day Radiation Therapy Episodes Over Time (2009-2020)

eFigure 3. Median Number of Radiation Fractions Overall and by Cancer Type, 2009 and 2020

eFigure 4. Adjusted Variation in Per-Practice Radiation Spending for All Cancer Patients Receiving RT, 2009 and 2020

eFigure 5. Adjusted Variation in Per-Practice Radiation Spending by Common Cancer Types

eFigure 6. Adjusted Variation in Per-Practice Fraction Number for All Cancer Patients Receiving Radiation Treatments

eFigure 7. Types of Radiation Technology by Common Cancer Types

eTable 6. Practice-Level Proportion of Only IMRT or Only Stereotactic RT Use Over Time

eTable 7. Number of Practices Utilizing Only IMRT or Only Stereotactic RT Over Time

This supplemental material has been provided by the authors to give readers additional information about their work.

© 2025 Lam MB et al. *JAMA Health Forum*.

eMethods

Standardized Spending

We used standardized spending to account for regional variation in payments made by Medicare across the United States. Specifically, for every radiation code used in the study (**eTable 2 and 3**), we determined the mean total payment (including both the payment from Medicare and payments by the patient or by other insurers) for each HCPCS/CPT code across all patients who received that treatment across all years. The mean spending amount for each code was then utilized for the remainder of the study. This standardization removes area-level differences in Medicare payments.

© 2025 Lam MB et al. *JAMA Health Forum*.

eTable 1. Cancer Diagnoses Codes

Cancer Types	ICD-9 Codes	ICD-10 Codes
Anal	154.2x, 154.3x	C21.xx
Bladder	188.xx	C67.xx
Bone Metastases	198.5x	C79.5x
Brain Metastases	198.3x	C79.3x
Breast	174.xx, 175.xx, 233.0x	C50.xx, D05.xx
Cervical	180.xx	C53.xx
Central Nervous System (CNS)	191.xx, 192.0x, 192.1x, 192.2x, 192.3x, 192.8x, 192.9x	C70.xx, C71.xx, C72.xx
Colorectal	153.xx, 154.0x, 154.1x, 154.8x	C18.xx, C19.xx, C20.xx
Head & Neck (H&N)	140.xx, 141.0x, 141.1x, 141.2x, 141.3x, 141.4x, 141.5x, 141.6x, 141.8x, 141.9x, 142.0x, 142.1x, 142.2x, 142.8x, 142.9x, 143.xx, 144.xx, 145.0x, 145.1x, 145.2x, 145.3x, 145.4x, 145.5x, 145.6x, 145.8x, 145.9x, 146.0x, 146.1x, 146.2x, 146.3x, 146.4x, 146.5x, 146.6x, 146.7x, 146.8x, 146.9x 147.xx, 148.0x, 148.1x, 148.2x, 148.3x, 148.8x, 148.9x, 149.xx, 160.0x, 160.1x, 160.2x, 160.3x, 160.4x, 160.5x, 160.8x, 160.9x, 161.xx, 195.0x	C00.xx, C01.xx, C02.xx, C03.xx, C04.xx, C05.xx, C06.xx, C07.xx, C08.xx, C09.xx, C10.xx, C11.xx, C12.xx, C13.xx, C14.xx, C30.xx, C31.xx, C32.xx, C76.0x
Lung	162.0x, 162.2x, 162.3x, 162.4x, 162.5x, 162.8x, 162.9x, 165.xx	C33.xx, C34.xx, C39.xx, C45.xx
Lymphoma	202.80, 202.81, 202.82, 202.83, 202.84, 202.85, 202.86, 202.87, 202.88, 203.80, 203.82, 200.0x, 200.1x, 200.2x, 200.3x, 200.4x, 200.5x, 200.6x, 200.7x, 200.8x, 201.xx, 202.0x, 202.1x, 202.2x, 202.4x, 202.7x, 273.3x	C81.xx, C82.xx, C83.xx, C84.xx, C85.xx, C86.xx, C88.xx, C91.4x
Pancreatic	157.xx	C25.xx
Prostate	185.xx	C61.xx
Upper Gastrointestinal	150.xx, 151.xx, 152.xx	C15.xx, C16.xx, C17.xx
Uterine	179.xx, 182.xx	C54.xx, C55.xx

© 2025 Lam MB et al. *JAMA Health Forum*.

eTable 2. Radiation Treatment HCPCS/CPT Codes to Define a Radiation Episode*

Description	HCPCS/CPT Codes
Simulation	77261, 77262, 77263
Radiation Delivery	77401, 77402, 77403, 77404, 77406, 77407, 77408, 77409, 77411, 77412, 77413, 77414, 77416, G6003, G6004, G6005, G6006, G6007, G6008, G6009, G6010, G6011, G6012, G6013, G6014, 0073T, 77385, 77386, 77418, G0174, G6015, G6016, 77371, 77372, 77373, G0173, G0243, G0251, G0339, G0340, 61796, 77520, 77521, 77522, 77523, 77524, 77525, 77761, 77762, 77764, 77767, 77768, 77770, 77771, 77772, 77778

*Modified from the episode identification specified by the formerly proposed CMS Radiation Oncology Model.

eTable 3. Radiation Treatment HCPCS/CPT Codes by Type of Technology

Description	HCPCS/CPT Codes
2D/3D	77401, 77402, 77403, 77404, 77406, 77407, 77408, 77409, 77411, 77412, 77413, 77414, 77416, G6003, G6004, G6005, G6006, G6007, G6008, G6009, G6010, G6011, G6012, G6013, G6014
IMRT	0073T, 77385, 77386, 77418, G0174, G6015, G6016
Stereotactic	77371, 77372, 77373, G0173, G0243, G0251, G0339, G0340, 61796
Proton	77520, 77521, 77522, 77523, 77524, 77525
Brachytherapy	77761, 77762, 77764, 77767, 77768, 77770, 77771, 77772, 77778
Codes not associated with one technology type	77261, 77262, 77263, 77014, 77021, 77280, 77285, 77290, 77293, 77295, 77299, 77300, 77301, 77306, 77307, 77321, 77331, 77332, 77333, 77334, 77336, 77338, 77370, 77399, 77417, 77427, 77431, 77432, 77435, 77470, 77499, G6017

*Each radiation episode was categorized into the type of technology used based on the predominant treatment method. If an episode utilized several technology types, the episode was assigned the technology that had the majority of fractions. Codes not associated with one technology type include, but are not limited to, simulation, respiratory motion management, and image guidance).

© 2025 Lam MB et al. *JAMA Health Forum*.

eTable 4. Cancer Patient Mortality during 90-Day Radiation Treatment Episodes

Cancer Types	Mortality Rate
All Cancer Types	13.8%
Anal	6.7%
Bladder	18.0%
Bone Metastases	30.0%
Brain Metastases	43.7%
Breast	1.6%
Cervical	8.7%
Central Nervous System (CNS)	25.0%
Colorectal	9.3%
Head & Neck (H&N)	8.4%
Lung	16.8%
Lymphoma	11.3%
Pancreatic	16.9%
Prostate	1.5%
Upper Gastrointestinal (GI)	18.8%
Uterine	5.4%
All Others	16.9%

eTable 5. Practice-level Adjusted Radiation Spending, Additional Models

Model		All Cancers	Prostate	Breast	Lung	Colo-rectal	Bone Metastasis	Brain Metastasis
	Mean	$13,683	$24,096	$11,427	$12,823	$12,946	$6,187	$8,193
**Model 1**	SD	$4,121	$4,601	$3,004	$2,647	$2,884	$2,046	$2,169
**Model 2**	SD	$2,756	$4,373	$2,996	$2,653	$2,886	$2,072	$2,165
**Model 3**	SD	$2,843	$4,469	$3,009	$2,610	$2,760	$2,053	$2,068
**Model 4**	SD	$1,781	$2,564	$1,836	$1,673	$1,455	$1,369	$1,334
**Model 5**	SD	$1,467	$2,064	$1,353	$1,388	$1,260	$1,046	$1,081
**Model 6**	SD	$1,444	$1,884	$1,349	$1,368	$1,224	$1,045	$1,082

Model 1: Year
Model 2: Year, age, sex, dual eligibility, hierarchical chronic conditions (HCC), cancer type
Model 3: Year, age, sex, dual eligibility, HCC, cancer type, hospital referral region (HRR)
Model 4: Year, age, sex, dual eligibility, HCC, cancer type, HRR, radiation technology type
Model 5: Year, age, sex, dual eligibility, HCC, cancer type, HRR, radiation technology type, number of fractions
Model 6: Year, age, sex, dual eligibility, HCC, cancer type, HRR, radiation technology type, number of fractions, freestanding vs hospital-based radiation facility.
Spending was calculated using standardized payments across all years.
SD=standard deviation

© 2025 Lam MB et al. *JAMA Health Forum*.

eFigure 1. Flowchart for Patient Population

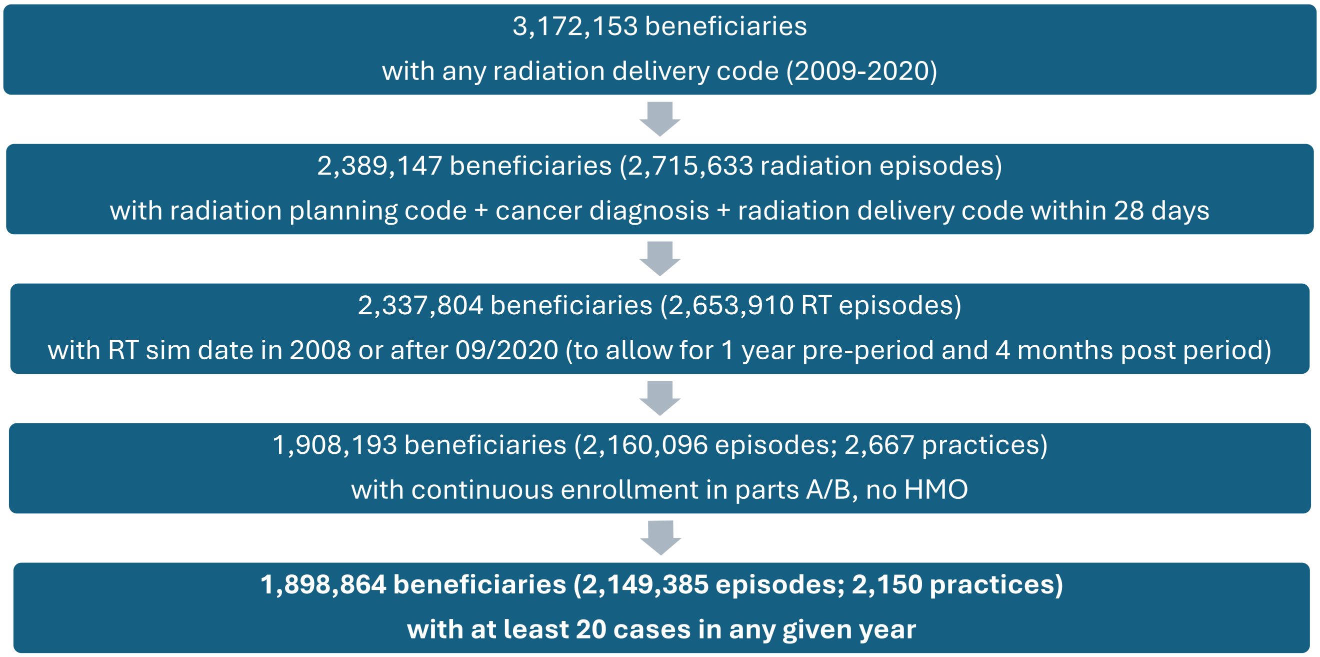

RT=radiation therapy, HMO=health maintenance organization

© 2025 Lam MB et al. *JAMA Health Forum*.

eFigure 2. Unadjusted Standardized Spending for Radiation Therapy-Specific Services for 90-Day Radiation Therapy Episodes Over Time (2009-2020)

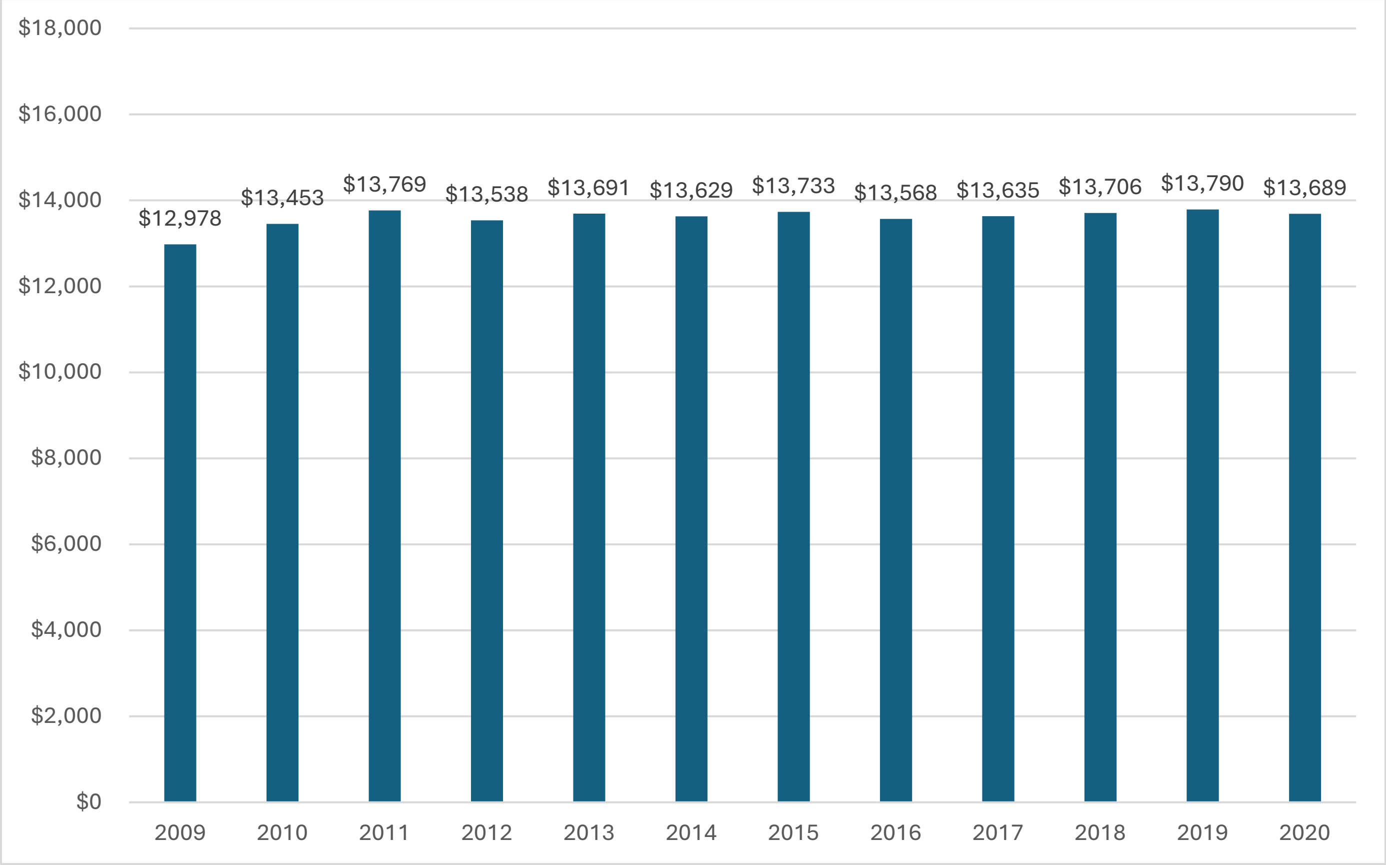

Spending was calculated using standardized payments across all years.

© 2025 Lam MB et al. *JAMA Health Forum*.

eFigure 3. Median Number of Radiation Fractions Overall and by Cancer Type, 2009 and 2020

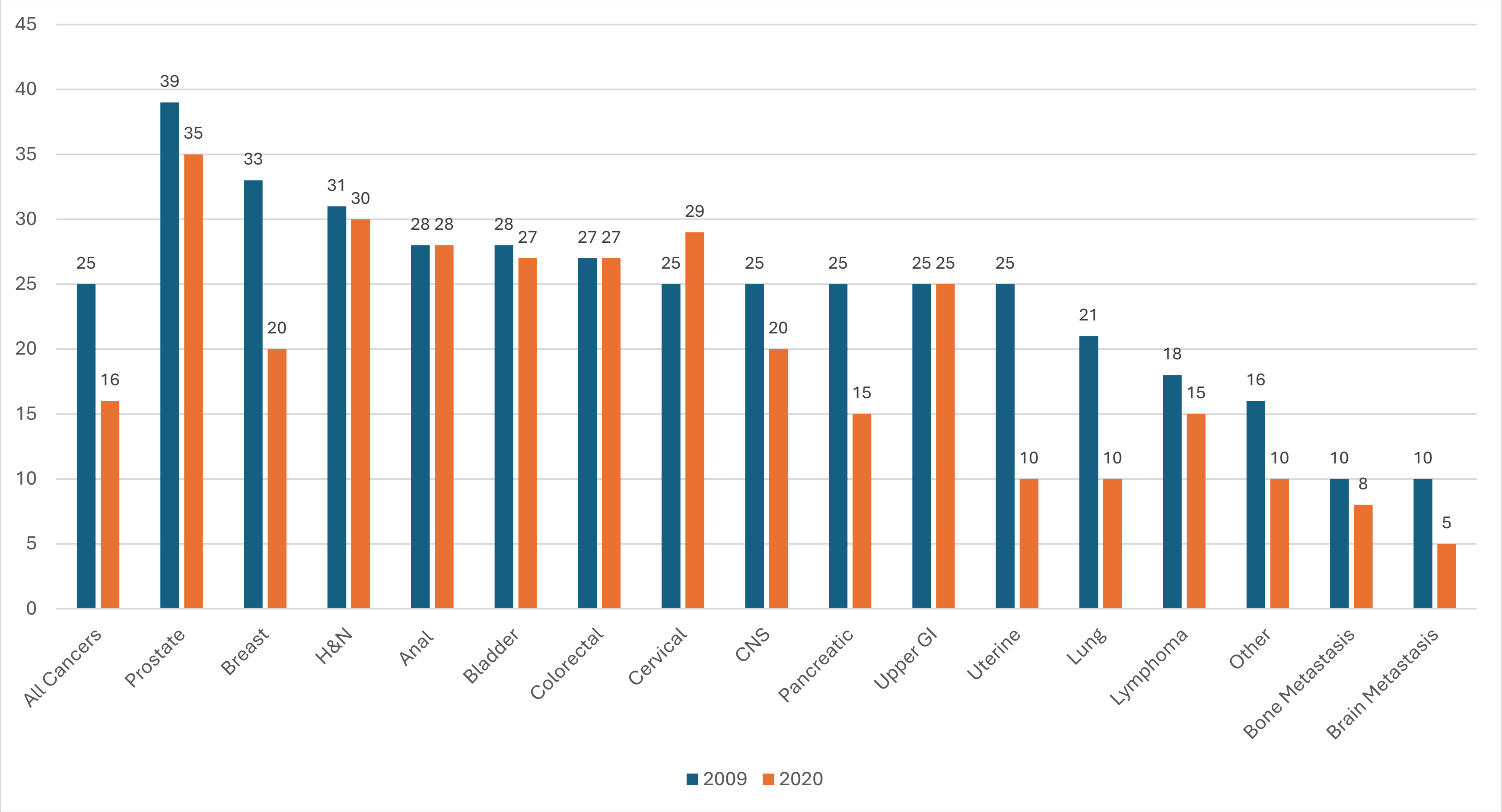

© 2025 Lam MB et al. *JAMA Health Forum*.

eFigure 4. Adjusted Variation in Per-Practice Radiation Spending for All Cancer Patients Receiving RT, 2009 and 2020

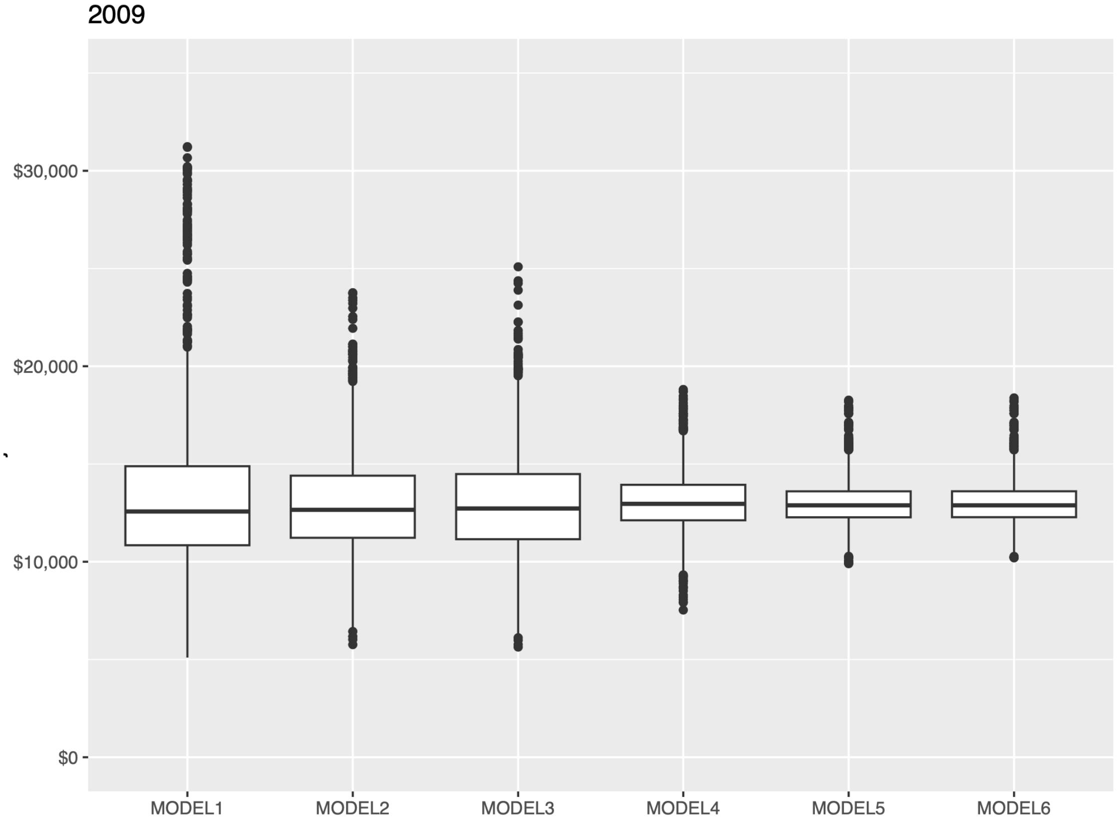

Model 1: Year
Model 2: Year, age, sex, dual eligibility, hierarchical chronic conditions (HCC), cancer type
Model 3: Year, age, sex, dual eligibility, HCC, cancer type, hospital referral region (HRR)
Model 4: Year, age, sex, dual eligibility, HCC, cancer type, HRR, radiation technology type
Model 5: Year, age, sex, dual eligibility, HCC, cancer type, HRR, radiation technology type, number of fractions
Model 6: Year, age, sex, dual eligibility, HCC, cancer type, HRR, radiation technology type, number of fractions, freestanding vs hospital-based radiation facility.
Outliers (<1%, >99%) were suppressed for the figure.
Spending was calculated using standardized payments across all years.

© 2025 Lam MB et al. *JAMA Health Forum*.

2020

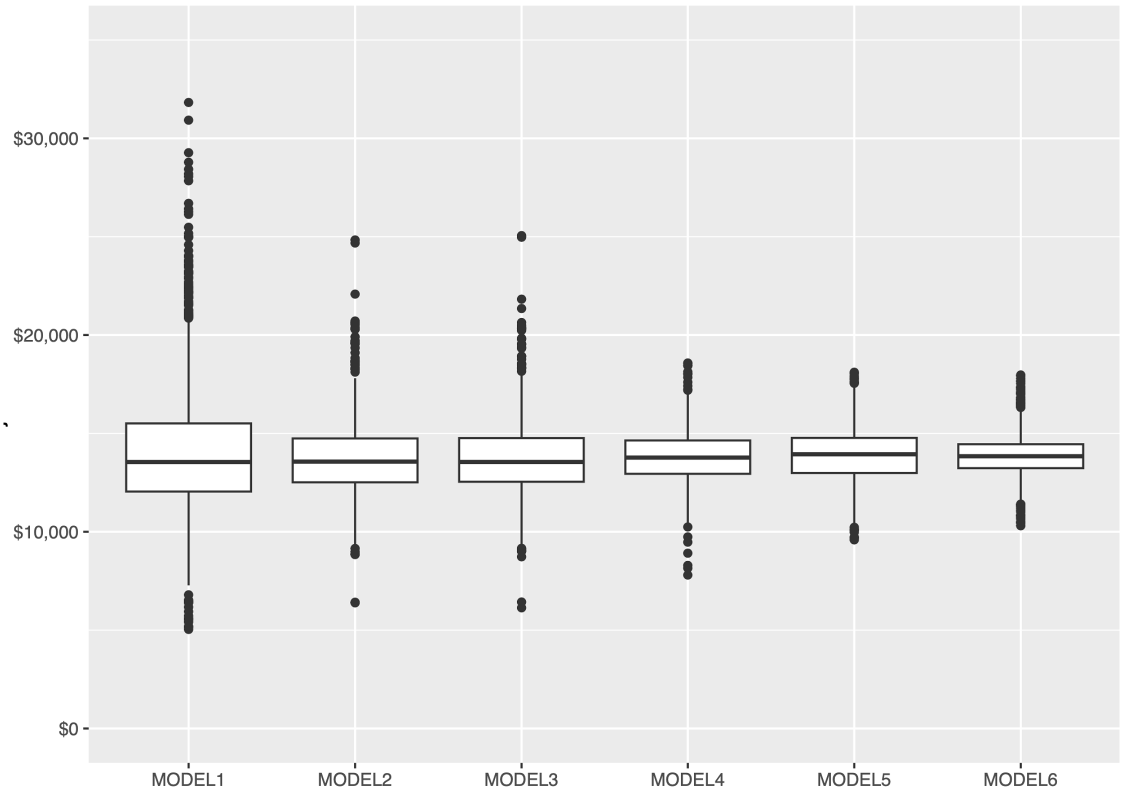

Model 1: Year
Model 2: Year, age, sex, dual eligibility, hierarchical chronic conditions (HCC), cancer type
Model 3: Year, age, sex, dual eligibility, HCC, cancer type, hospital referral region (HRR)
Model 4: Year, age, sex, dual eligibility, HCC, cancer type, HRR, radiation technology type
Model 5: Year, age, sex, dual eligibility, HCC, cancer type, HRR, radiation technology type, number of fractions
Model 6: Year, age, sex, dual eligibility, HCC, cancer type, HRR, radiation technology type, number of fractions, freestanding vs hospital-based radiation facility.
Outliers (<1%, >99%) were suppressed for the figure.
Spending was calculated using standardized payments across all years.

© 2025 Lam MB et al. *JAMA Health Forum*.

eFigure 5. Adjusted Variation in Per-Practice Radiation Spending by Common Cancer Types

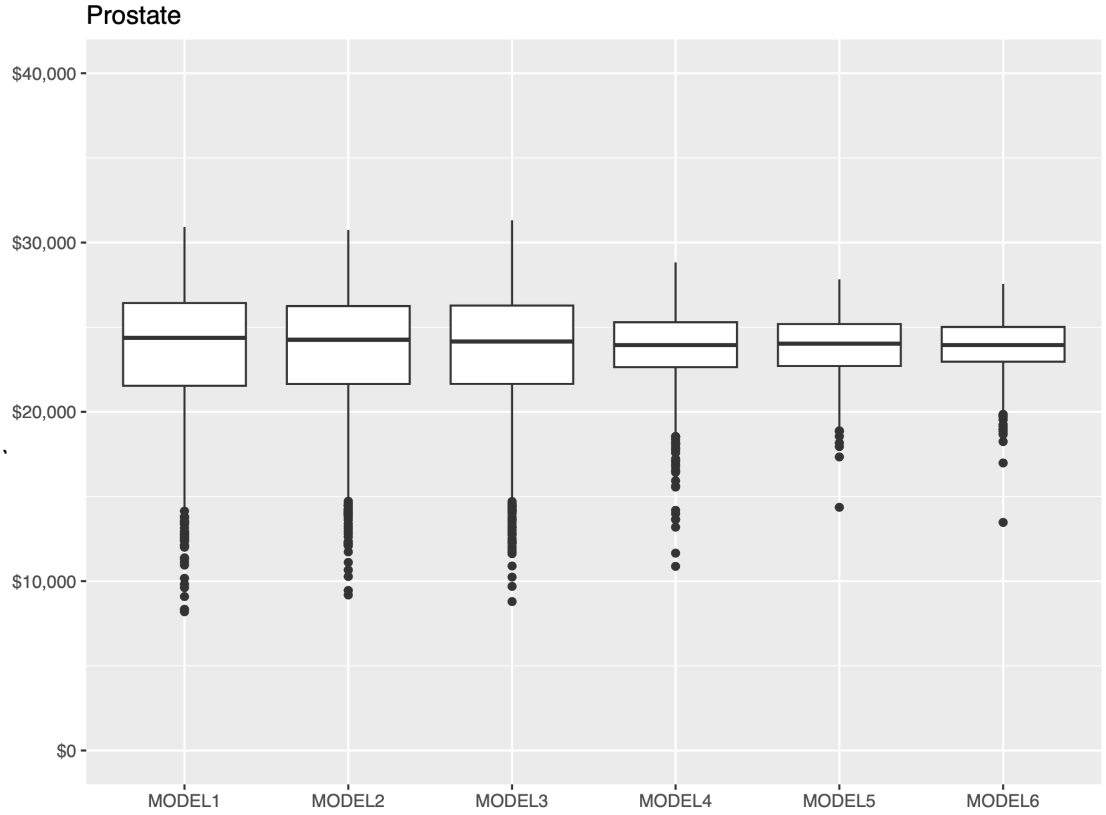

Model 1: Year
Model 2: Year, age, sex, dual eligibility, hierarchical chronic conditions (HCC), cancer type
Model 3: Year, age, sex, dual eligibility, HCC, cancer type, hospital referral region (HRR)
Model 4: Year, age, sex, dual eligibility, HCC, cancer type, HRR, radiation technology type
Model 5: Year, age, sex, dual eligibility, HCC, cancer type, HRR, radiation technology type, number of fractions
Model 6: Year, age, sex, dual eligibility, HCC, cancer type, HRR, radiation technology type, number of fractions, freestanding vs hospital-based radiation facility.
Outliers (<1%, >99%) were suppressed for the figure.
Spending was calculated using standardized payments across all years.

© 2025 Lam MB et al. *JAMA Health Forum*.

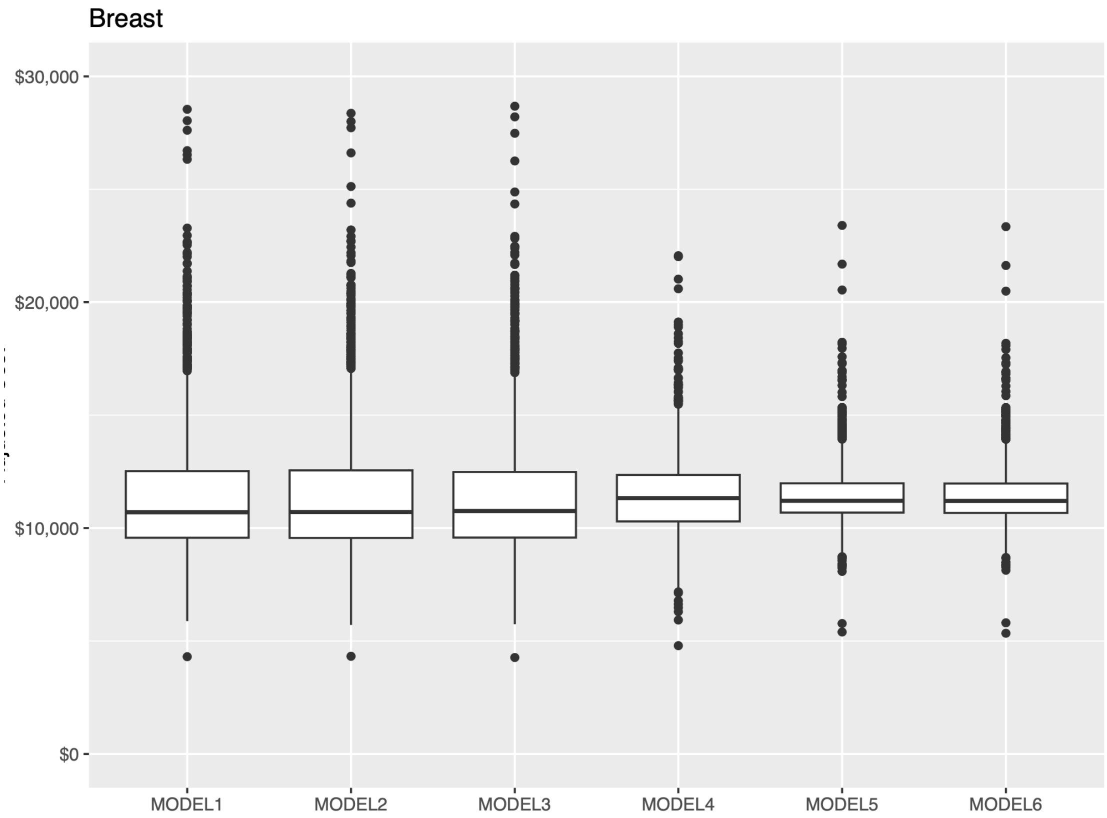

Model 1: Year
Model 2: Year, age, sex, dual eligibility, hierarchical chronic conditions (HCC), cancer type
Model 3: Year, age, sex, dual eligibility, HCC, cancer type, hospital referral region (HRR)
Model 4: Year, age, sex, dual eligibility, HCC, cancer type, HRR, radiation technology type
Model 5: Year, age, sex, dual eligibility, HCC, cancer type, HRR, radiation technology type, number of fractions
Model 6: Year, age, sex, dual eligibility, HCC, cancer type, HRR, radiation technology type, number of fractions, freestanding vs hospital-based radiation facility.
Outliers (<1%, >99%) were suppressed for the figure.
Spending was calculated using standardized payments across all years.

© 2025 Lam MB et al. *JAMA Health Forum*.

Lung

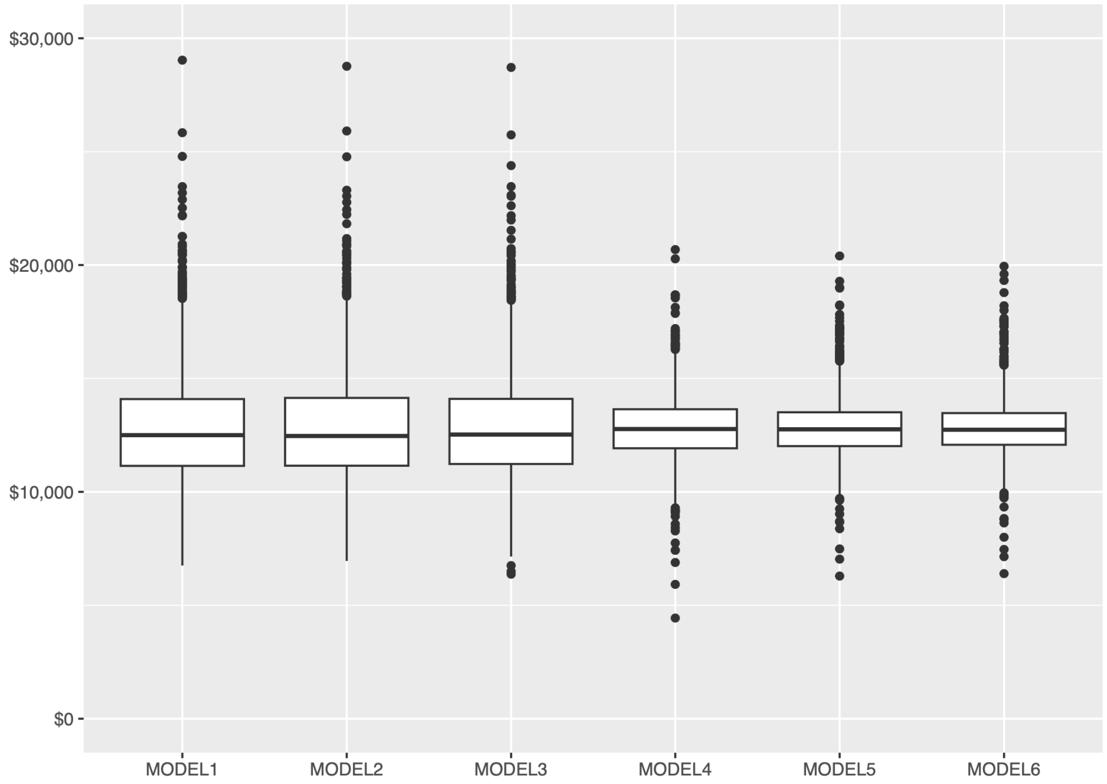

Model 1: Year
Model 2: Year, age, sex, dual eligibility, hierarchical chronic conditions (HCC), cancer type
Model 3: Year, age, sex, dual eligibility, HCC, cancer type, hospital referral region (HRR)
Model 4: Year, age, sex, dual eligibility, HCC, cancer type, HRR, radiation technology type
Model 5: Year, age, sex, dual eligibility, HCC, cancer type, HRR, radiation technology type, number of fractions
Model 6: Year, age, sex, dual eligibility, HCC, cancer type, HRR, radiation technology type, number of fractions, freestanding vs hospital-based radiation facility.
Outliers (<1%, >99%) were suppressed for the figure.
Spending was calculated using standardized payments across all years.

© 2025 Lam MB et al. *JAMA Health Forum*.

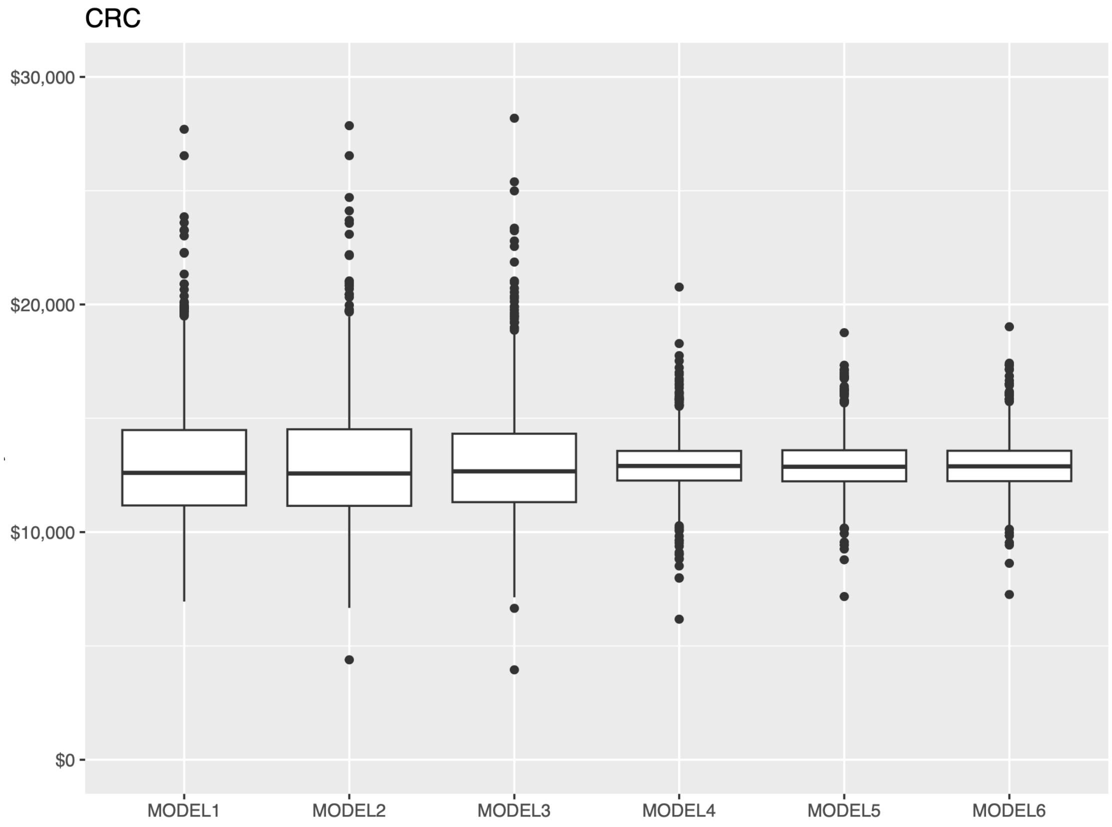

Model 1: Year
Model 2: Year, age, sex, dual eligibility, hierarchical chronic conditions (HCC), cancer type
Model 3: Year, age, sex, dual eligibility, HCC, cancer type, hospital referral region (HRR)
Model 4: Year, age, sex, dual eligibility, HCC, cancer type, HRR, radiation technology type
Model 5: Year, age, sex, dual eligibility, HCC, cancer type, HRR, radiation technology type, number of fractions
Model 6: Year, age, sex, dual eligibility, HCC, cancer type, HRR, radiation technology type, number of fractions, freestanding vs hospital-based radiation facility.
Outliers (<1%, >99%) were suppressed for the figure.
Spending was calculated using standardized payments across all years.

© 2025 Lam MB et al. *JAMA Health Forum*.

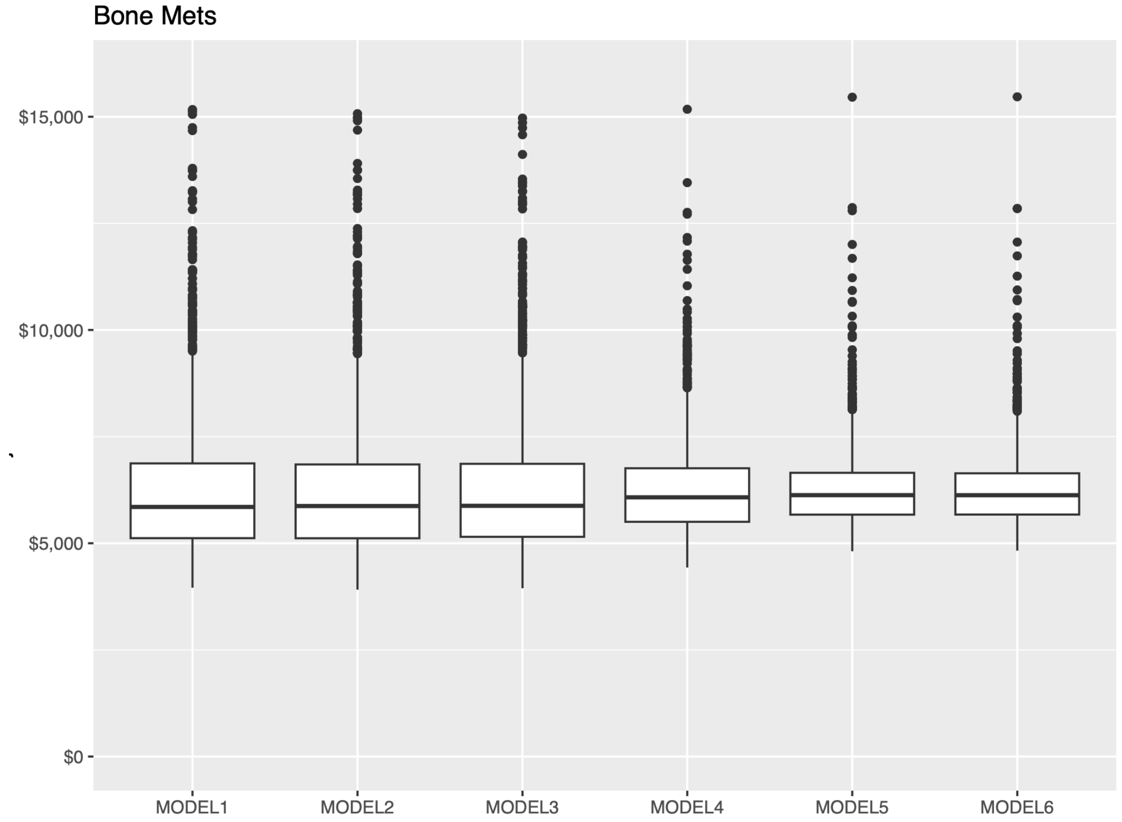

Model 1: Year
Model 2: Year, age, sex, dual eligibility, hierarchical chronic conditions (HCC), cancer type
Model 3: Year, age, sex, dual eligibility, HCC, cancer type, hospital referral region (HRR)
Model 4: Year, age, sex, dual eligibility, HCC, cancer type, HRR, radiation technology type
Model 5: Year, age, sex, dual eligibility, HCC, cancer type, HRR, radiation technology type, number of fractions
Model 6: Year, age, sex, dual eligibility, HCC, cancer type, HRR, radiation technology type, number of fractions, freestanding vs hospital-based radiation facility.
Outliers (<1%, >99%) were suppressed for the figure.
Spending was calculated using standardized payments across all years.

© 2025 Lam MB et al. *JAMA Health Forum*.

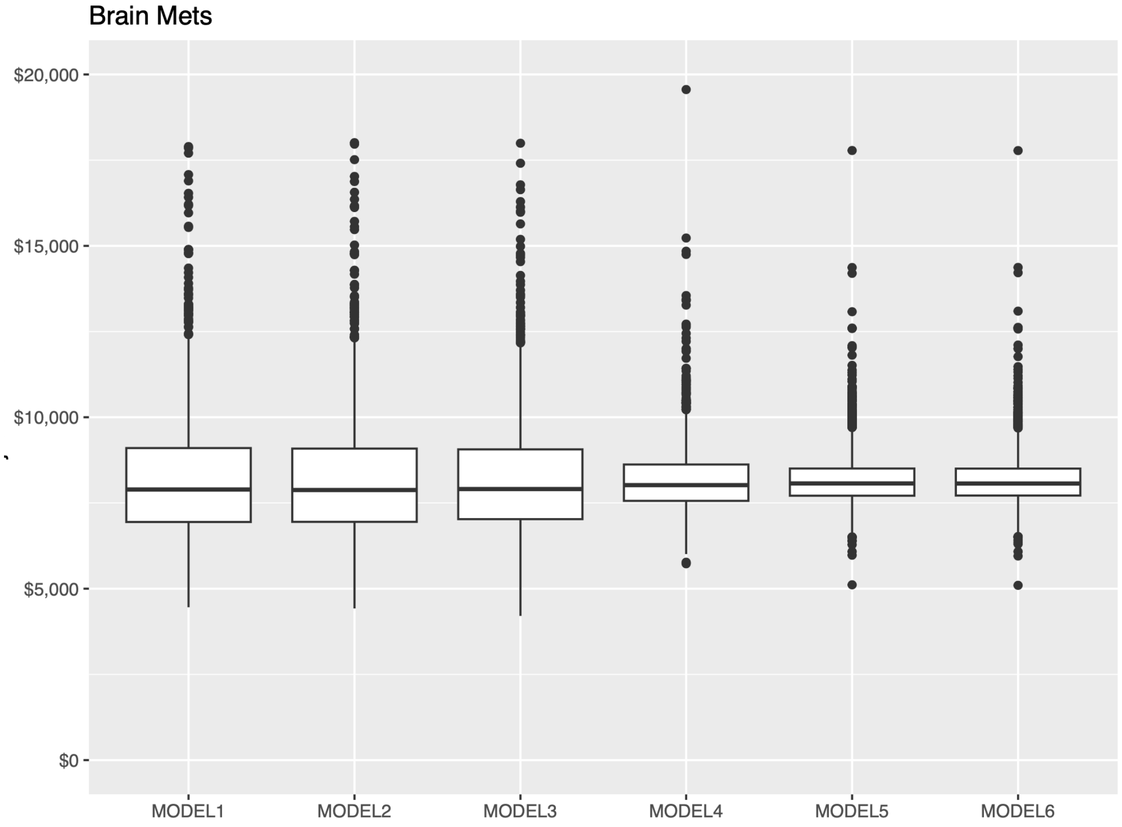

Model 1: Year
Model 2: Year, age, sex, dual eligibility, hierarchical chronic conditions (HCC), cancer type
Model 3: Year, age, sex, dual eligibility, HCC, cancer type, hospital referral region (HRR)
Model 4: Year, age, sex, dual eligibility, HCC, cancer type, HRR, radiation technology type
Model 5: Year, age, sex, dual eligibility, HCC, cancer type, HRR, radiation technology type, number of fractions
Model 6: Year, age, sex, dual eligibility, HCC, cancer type, HRR, radiation technology type, number of fractions, freestanding vs hospital-based radiation facility.
Outliers (<1%, >99%) were suppressed for the figure.
Spending was calculated using standardized payments across all years.

© 2025 Lam MB et al. *JAMA Health Forum*.

eFigure 6. Adjusted Variation in Per-Practice Fraction Number for All Cancer Patients Receiving Radiation Treatments

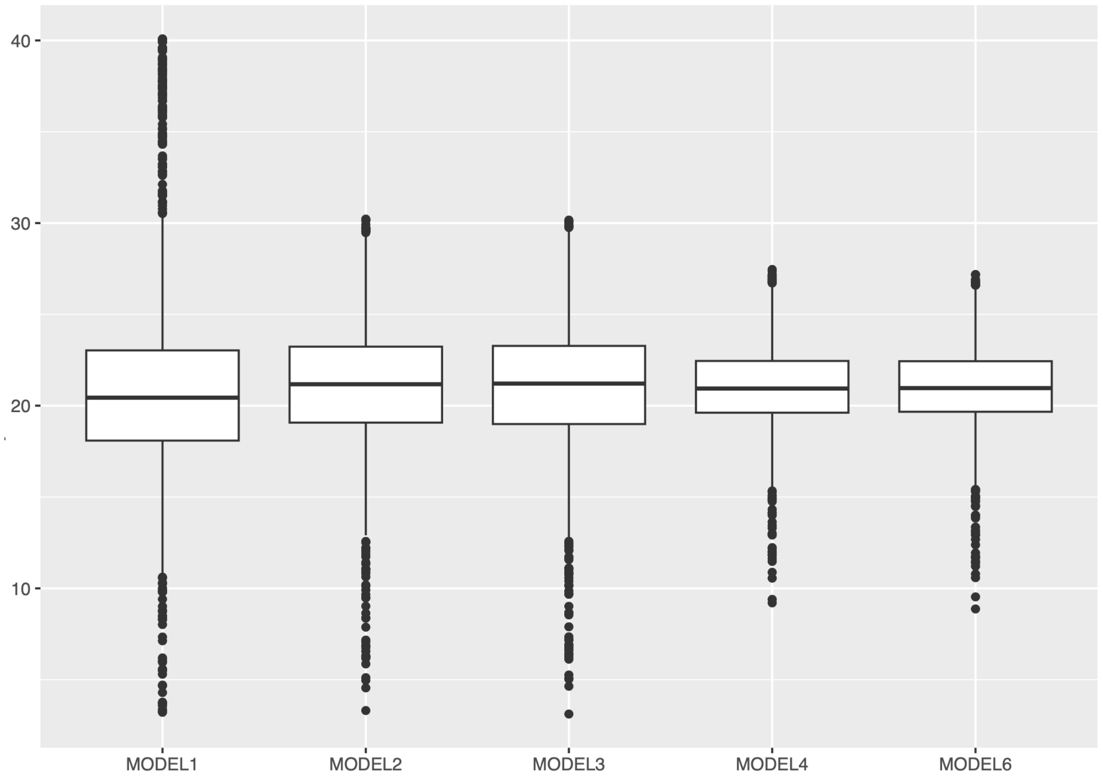

Model 1: Year
Model 2: Year, age, sex, dual eligibility, hierarchical chronic conditions (HCC), cancer type
Model 3: Year, age, sex, dual eligibility, HCC, cancer type, hospital referral region (HRR)
Model 4: Year, age, sex, dual eligibility, HCC, cancer type, HRR, radiation technology type
Model 5: Year, age, sex, dual eligibility, HCC, cancer type, HRR, radiation technology type, number of
fractions
Model 6: Year, age, sex, dual eligibility, HCC, cancer type, HRR, radiation technology type, number of
fractions, freestanding vs hospital-based radiation facility.
Outliers (<1%, >99%) were suppressed for the figure.
Spending was calculated using standardized payments across all years.

© 2025 Lam MB et al. *JAMA Health Forum*.

eFigure 7. Types of Radiation Technology by Common Cancer Types

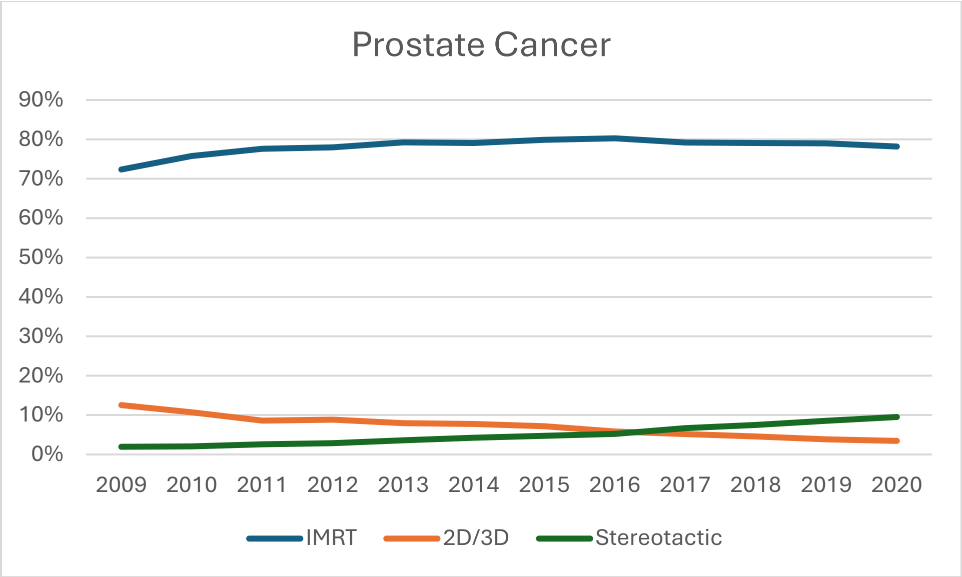

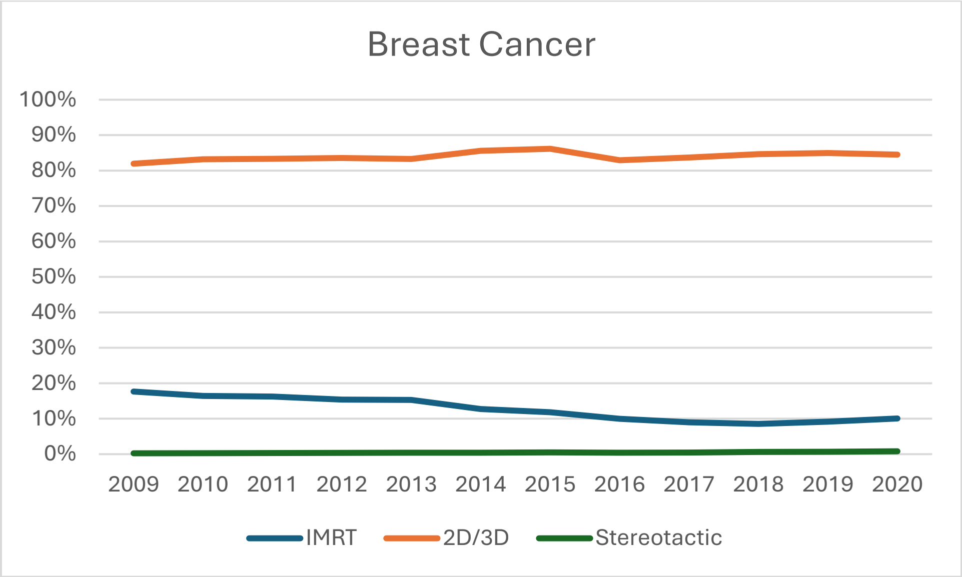

© 2025 Lam MB et al. *JAMA Health Forum*.

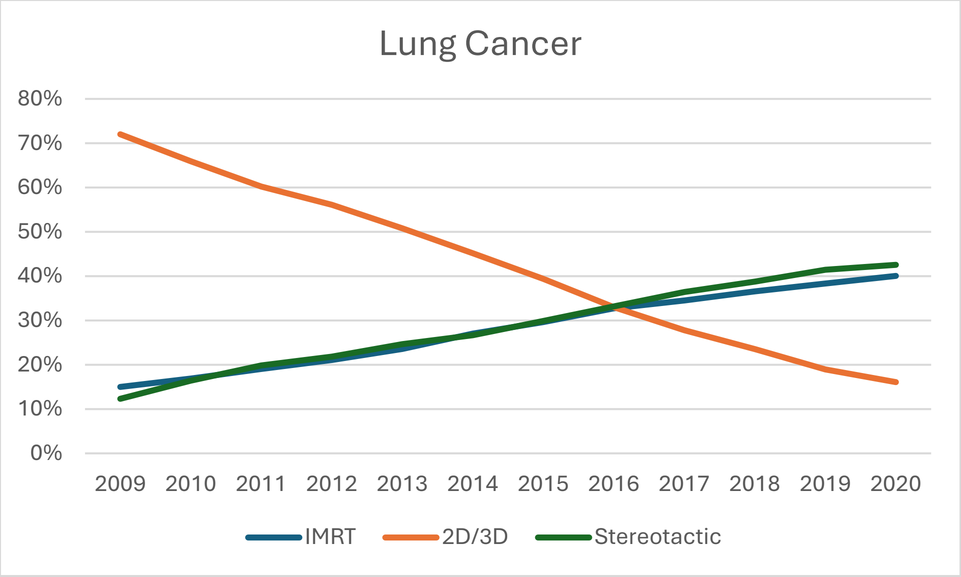

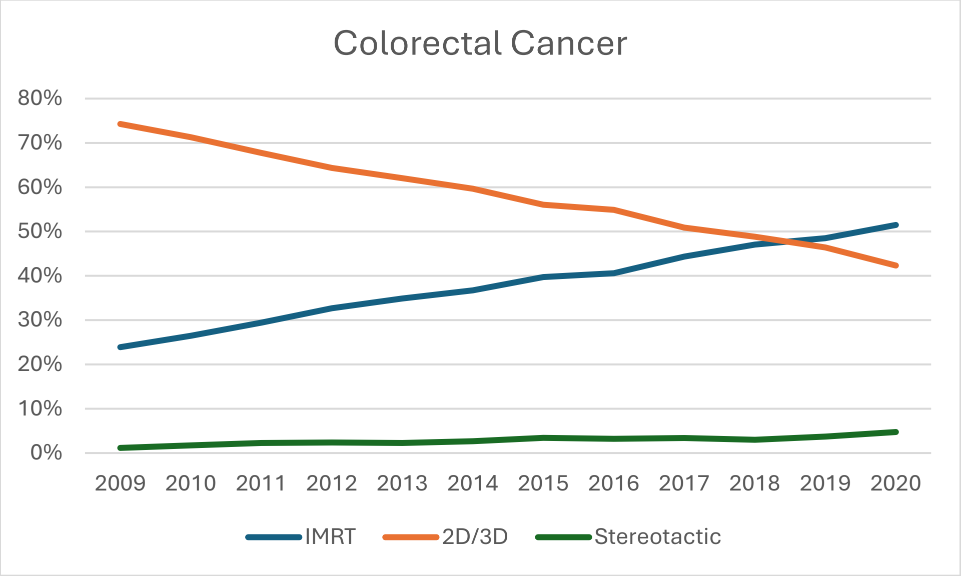

© 2025 Lam MB et al. *JAMA Health Forum*.

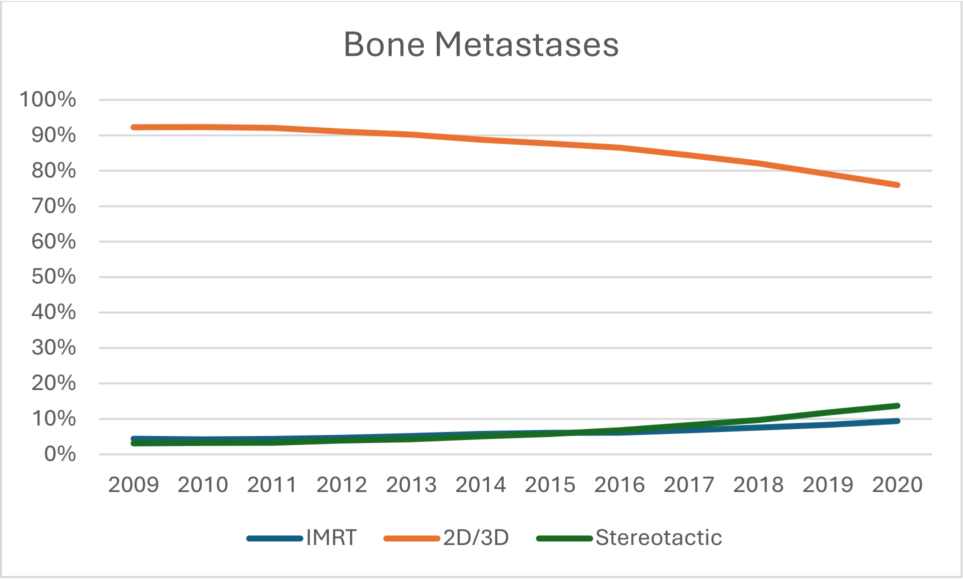

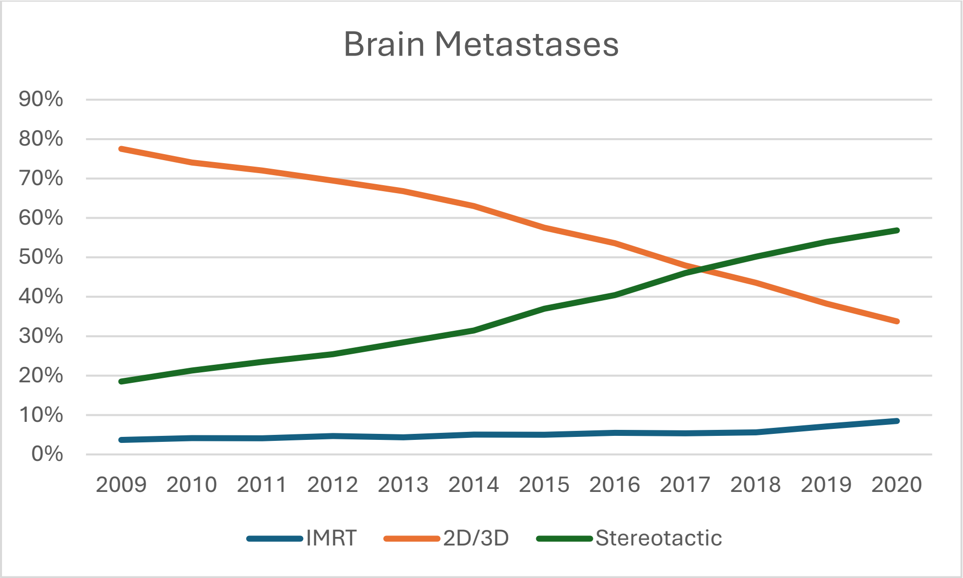

Brachytherapy and proton radiation are not allowed to be reported by CMS due to small numbers.

© 2025 Lam MB et al. *JAMA Health Forum*.

eTable 6. Practice-Level Proportion of Only IMRT or Only Stereotactic RT Use Over Time

IMRT					
**Year**	**Min**	**25th%**	**Median**	**75th%**	**Max**
2009	0%	17%	28%	43%	100%
2010	0%	18%	28%	44%	100%
2011	0%	21%	31%	46%	100%
2012	0%	20%	31%	45%	100%
2013	0%	22%	32%	46%	100%
2014	0%	23%	33%	46%	100%
2015	0%	24%	35%	47%	100%
2016	0%	26%	36%	47%	100%
2017	0%	28%	36%	48%	100%
2018	0%	30%	38%	49%	100%
2019	0%	31%	39%	50%	100%
2020	0%	33%	40%	50%	100%
**Stereotactic**					
**Year**	**Min**	**25th%**	**Median**	**75th%**	**Max**
2009	0%	0%	0%	4%	100%
2010	0%	0%	0%	5%	100%
2011	0%	0%	0%	7%	100%
2012	0%	0%	2%	8%	100%
2013	0%	0%	3%	10%	100%
2014	0%	0%	4%	11%	100%
2015	0%	0%	6%	13%	100%
2016	0%	0%	7%	14%	100%
2017	0%	0%	9%	15%	100%
2018	0%	2%	10%	18%	100%
2019	0%	4%	11%	18%	100%
2020	0%	6%	13%	20%	100%

eTable 7. Number of Practices Utilizing Only IMRT or Only Stereotactic RT Over Time

	**2009**	**2010**	**2011**	**2012**	**2013**	**2014**	**2015**	**2016**	**2017**	**2018**	**2019**	**2020**
**IMRT**	29	26	26	17	9	14	11	13	11	14	12	7
**Stereotactic**	3	3	1	1	3	2	2	1	1	1	1	1

*Practices utilizing 100% IMRT were focused on treating urologic cancers and practices utilizing 100% stereotactic RT were focused on treating urologic and lung cancers.

© 2025 Lam MB et al. *JAMA Health Forum*.
