# Supplementary material for: Practice-Level Spending Variation for Radiation Treatment Episodes Among Older Adults With Cancer
Source: JAMA Health Forum. 2025 Jul 18;6(7):e251952. doi: 10.1001/jamahealthforum.2025.1952 (PMC12274980; doi:10.1001/jamahealthforum.2025.1952)
Supplement: Supplement 2. — Data Sharing Statement [file jamahealthforum-e251952-s002.pdf]

Data Sharing Statement

Lam. Practice-Level Spending Variation for Radiation Treatment Episodes Among Older Adults With Cancer. *JAMA Health Forum*. Published July 18, 2025.
doi:10.1001/jamahealthforum.2025.1952

Data
Data available: No

Additional Information
Explanation for why data not available: Centers for Medicare and Medicaid (CMS) does not allow for direct sharing of data to protect patient privacy and confidentiality. Patient data can be accessed through CMS.
